# A network of acetyl phosphate-dependent modification modulates c-di-AMP homeostasis in *Actinobacteria*

**DOI:** 10.1128/mbio.01411-24

**Published:** 2024-07-09

**Authors:** Yu Fu, Liu-Chang Zhao, Jin-Long Shen, Shi-Yu Zhou, Bin-Cheng Yin, Bang-Ce Ye, Di You

**Affiliations:** 1 Laboratory of Biosystems and Microanalysis, State Key Laboratory of Bioreactor Engineering, East China University of Science and Technology, Shanghai, China; 2 Institute of Engineering Biology and Health, Collaborative Innovation Center of Yangtze River Delta Region Green Pharmaceuticals, College of Pharmaceutical Sciences, Zhejiang University of Technology, Hangzhou, Zhejiang, China; The University of Texas Health Science Center at Houston, Houston, Texas, USA

**Keywords:** AcP, c-di-AMP, DisA, acetylation, *Actinobacteria*

## Abstract

Cyclic purine nucleotides are important signal transduction molecules across all domains of life. 3′,5′-cyclic di-adenosine monophosphate (c-di-AMP) has roles in both prokaryotes and eukaryotes, while the signals that adjust intracellular c-di-AMP and the molecular machinery enabling a network-wide homeostatic response remain largely unknown. Here, we present evidence for an acetyl phosphate (AcP)-governed network responsible for c-di-AMP homeostasis through two distinct substrates, the diadenylate cyclase DNA integrity scanning protein (DisA) and its newly identified transcriptional repressor, DasR. Correspondingly, we found that AcP-induced acetylation exerts these regulatory actions by disrupting protein multimerization, thus impairing c-di-AMP synthesis via K66 acetylation of DisA. Conversely, the transcriptional inhibition of *disA* was relieved during DasR acetylation at K78. These findings establish a pivotal physiological role for AcP as a mediator to balance c-di-AMP homeostasis. Further studies revealed that acetylated DisA and DasR undergo conformational changes that play crucial roles in differentiation. Considering the broad distribution of AcP-induced acetylation in response to environmental stress, as well as the high conservation of the identified key sites, we propose that this unique regulation of c-di-AMP homeostasis may constitute a fundamental property of central circuits in *Actinobacteria* and thus the global control of cellular physiology.

**IMPORTANCE:**

Since the identification of c-di-AMP is required for bacterial growth and cellular physiology, a major challenge is the cell signals and stimuli that feed into the decision-making process of c-di-AMP concentration and how that information is integrated into the regulatory pathways. Using the bacterium *Saccharopolyspora erythraea* as a model, we established that AcP-dependent acetylation of the diadenylate cyclase DisA and its newly identified transcriptional repressor DasR is involved in coordinating environmental and intracellular signals, which are crucial for c-di-AMP homeostasis. Specifically, DisA acetylated at K66 directly inactivates its diadenylate cyclase activity, hence the production of c-di-AMP, whereas DasR acetylation at K78 leads to increased *disA* expression and c-di-AMP levels. Thus, AcP represents an essential molecular switch in c-di-AMP maintenance, responding to environmental changes and possibly hampering efficient development. Therefore, AcP-mediated posttranslational processes constitute a network beyond the usual and well-characterized synthetase/hydrolase governing c-di-AMP homeostasis.

## INTRODUCTION

Since its discovery in 2008, the focus and interest surrounding the second messenger 3′,5′-cyclic di-adenosine monophosphate (c-di-AMP) have been steadily increasing. c-di-AMP is widespread among bacteria and archaea, wherein many species require c-di-AMP for growth and survival (1–4). It is primarily involved in regulating various biological processes, particularly those related to maintaining cell wall integrity and osmotic balance (5–10). Moreover, mounting evidence suggests that some c-di-AMP-synthesizing organisms are prominent human pathogens and are of environmental importance (11–17).

The maintenance and stability of c-di-AMP are fundamental to c-di-AMP-producing species. Elevated c-di-AMP levels result in aberrant physiology, given the expanding number of recognized organisms that synthesize c-di-AMP and the importance it plays in the diverse environments that bacteria encounter (18). Similar to other second messengers, c-di-AMP levels must be controlled to prevent toxic accumulation. These depletion strategies appear to be regulated by environmental and intracellular signals and are integrated with other stress response pathways. Upon environmental stimulation, changes in intracellular c-di-AMP levels rely on the activity of diadenylate cyclase (DAC) domain-containing proteins or c-di-AMP-specific phosphodiesterase (10). The DAC domain was first identified in the DNA integrity scanning protein (DisA) (19). To date, four classes of DACs have been characterized—DisA, CdaA, CdaS, and CdaM—while most organisms contain only one type of DAC (3).

Despite significant progress in our understanding of c-di-AMP synthesis and hydrolysis, the signals that adjust the intracellular c-di-AMP concentration and how this information is converted into feedback signaling, as well as the regulatory mechanism behind its synthesis and degradation, remain largely unknown. In bacteria, DAC domain proteins are most frequently found in the Gram-positive *Firmicutes* and within the phylum *Actinobacteria* (2). *Actinobacteria* are ubiquitous, primarily soil-dwelling bacteria with a complicated developmental life cycle involving the progression from vegetative growth to the production of reproductive aerial hyphae that differentiate into chains of exospores (20). Entry into development coincides with the biosynthesis of numerous secondary metabolites that serve as the most abundant source of clinically important antibiotics and provide other medically important drugs, for instance, anti-cancer agents and immunosuppressants (21–23). Consequently, there is considerable interest in understanding the mechanism that relate to this developmental transition.

Recent work on protein acetylation in Gram-positive *Actinobacteria* has highlighted the importance of acetylation as a signal that provides functional diversity and regulation by modifying proteins to respond to diverse stimuli, which provides a direct link between cell metabolism and signal transduction, transcriptional regulation, cell growth, and pathogenicity (24, 25). Acetylation is one of many posttranslational modifications (PTMs) that are important in biological systems (26). Bacterial acetylation depends on both enzymatic and nonenzymatic mechanisms of acetylation, and both mechanisms can control the properties of that protein, such as enzymatic activity, localization, stability, or interactions with other molecules (27, 28).

Here, we show that c-di-AMP maintenance is processed by a network of acetyl phosphate (AcP)-induced acetylation. Acetylation induced by AcP disrupts the multimerization of its substrates in *Saccharopolyspora erythraea*, leading to impaired c-di-AMP synthesis via K66 acetylation of DisA. Conversely, acetylation of DasR at K78 relieves the transcriptional inhibition of *disA* and promotes intracellular c-di-AMP levels, altogether supporting c-di-AMP homeostasis. Altered c-di-AMP levels either through DisA or DasR acetylation could cause a metabolic imbalance, leading to an alert signal for developmental transitions. These findings highlight the crucial role of AcP in balancing c-di-AMP levels and suggest that this unique regulation is likely a necessary feature in *Actinobacteria*.

## RESULTS

### AcP-dependent acetylation directly impairs the DAC activity of DisA

**Fig 1:**
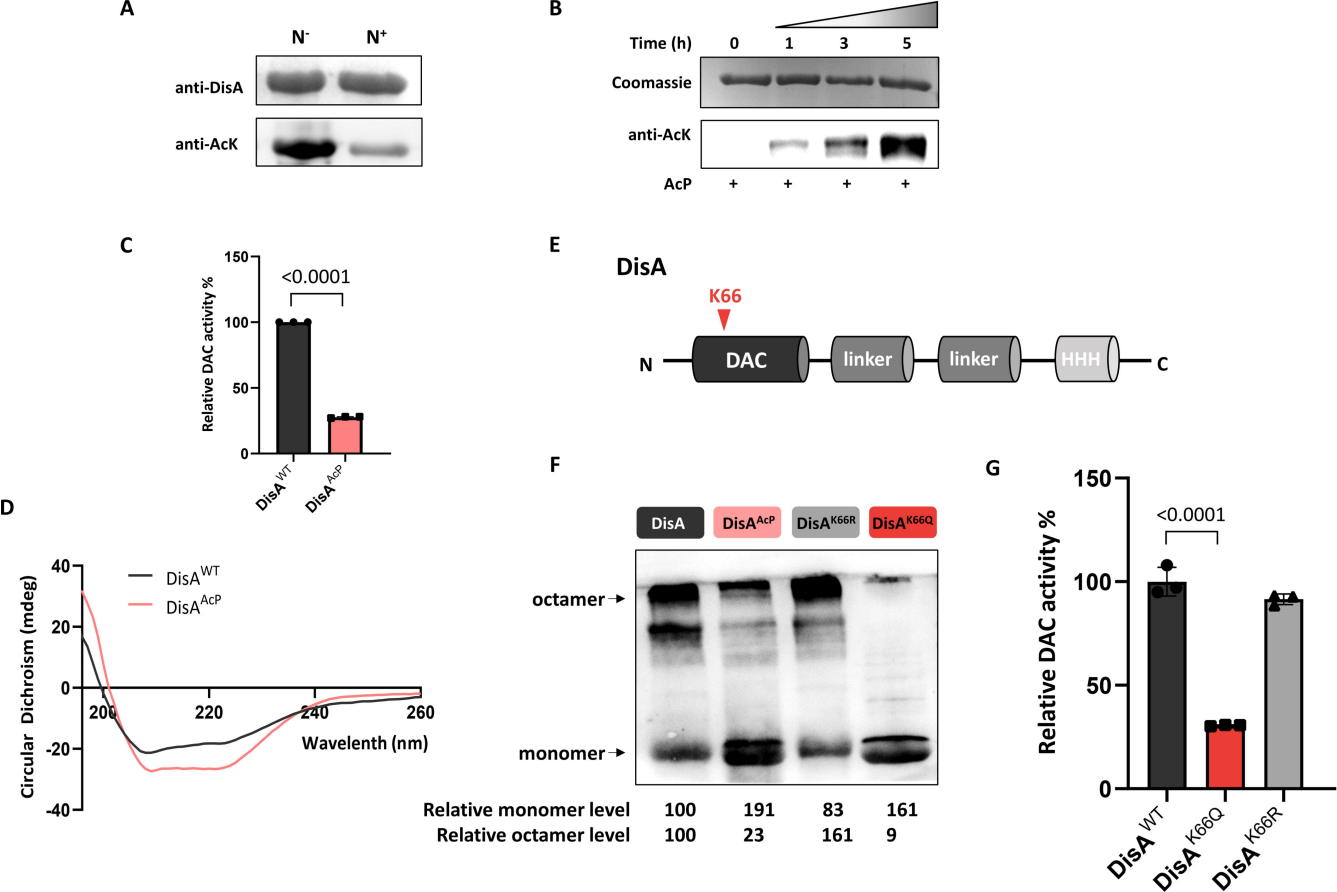
AcP-induced acetylation of DisA impairs its DAC activity. (**A**) Acetylation level of DisA from the *S. erythraea* wild type (WT) strain under excess nitrogen (N^+^) or limited nitrogen (N^−^) conditions. Each lane was loaded with an equal amount of DisA protein. (**B**) *In vitro* acetylation of His-tagged DisA protein with 10-mM AcP for various lengths of time (0, 1, 3, and 5 h) at 37°C. Acetylation levels were determined by Western blotting with a specific anti-AcK antibody. (**C**) DAC activity of the DisA^WT^ and DisA^AcP^ proteins. The DAC activity of DisA^WT^ was set to 100%. (**D**) CD spectra of the DisA^WT^ and DisA^AcP^ proteins. (**E**) Domain organization of the DisA is shown in differently colored boxes. The red inverted triangle shows the position of K66. (**F**) Cross-linking of the DisA^WT^, DisA^AcP^, DisA^K66Q^, and DisA^K66R^ proteins. The band intensities were quantified by densitometry using ImageJ software. The relative densitometry of the monomer and octamer in the DisA was set to 100. (**G**) DAC activity of the DisA^WT^, DisA^K66Q^, and DisA^K66R^ proteins. The DAC activity of DisA^WT^ was set to 100%. The error bars show the SDs of three independent experiments. Ordinary one-way analysis of variance was used for the statistical test.

Our previous study revealed that nitrogen starvation leads to AcP accumulation in *S. erythraea*, thus exerting a general effect on the global acetylation level in an AcP-mediated manner (24). The *S. erythraea* genome encodes only one type of DAC (DisA, SACE_0435) (Kyoto Encyclopedia of Genes and Genomes database, http://www.kegg.jp/). To explore the role of AcP in intracellular c-di-AMP homeostasis, we first tested whether DisA is a substrate of acetylation in Gram-positive *S. erythraea*. The acetylation status of DisA *in vivo* was tested by immunoprecipitation (IP) and immunoblotting (IB) analyses. DisA from *S. erythraea* cells was immunoprecipitated with an antibody against DisA. Acetyl-lysine levels were detected in DisA immunoprecipitates with an anti-AcK antibody. As shown in Fig. 1A, DisA was hyperacetylated under nitrogen starvation conditions. *In vitro* acetylation also showed that DisA could be acetylated by AcP (Fig. 1B). These results demonstrated that the DisA protein is a new acetylation substrate both *in vitro* and *in vivo*. To investigate the effect of acetylation on the DAC activity of DisA, DisA was incubated with or without AcP for 5 h. The activities of nonacetylated DisA (DisA^WT^) and acetylated DisA (DisA^AcP^) enzymes were determined. DisA^AcP^ activity was reduced by ∼75%, indicating that AcP-dependent acetylation effectively decreased its DAC activity (Fig. 1C). DisA forms a large octamer that possesses diadenylate cyclase activity (19). The secondary structure of DisA was then examined by circular dichroism (CD) assay. The far-UV CD spectra showed that AcP-dependent acetylation caused an influence on the DisA structure with lower α-helicity and a concomitant increase in anti-parallel and random coil structures compared with DisA^WT^ (Fig. 1D; Table S1). These results suggested that AcP-dependent acetylation likely plays a key role in the synthesis of c-di-AMP.

### DisA is inactive mainly upon acetylation of K66

To determine the acetylation sites of DisA, the *in vitro* and *in vivo* acetylated DisA proteins were analyzed by liquid chromatography-tandem mass spectrometry (LC-MS/MS). As listed in Table S2 and Fig. S1 through S3, two acetylated peptides containing K66 and K284 were identified in AcP-dependent acetylated DisA, while only K66 was identified in endogenous DisA. K66 is located in the DAC domain, which mediates diadenylate cyclase activity (Fig. 1E). To examine the K66 acetylation effect in detail, we introduced substitutions at the acetylated site K66 to generate the variants DisA^K66Q^ and DisA^K66R^ based on the principle that glutamine (Q) can serve as a structural mimic for acetyl-lysine and that arginine (R) serves as a genetic mimic of unacetylated lysine (29). The DisA^WT^, DisA^AcP^, DisA^K66Q^, and DisA^K66R^ proteins were then subjected to both chemical cross-linking and DAC assays, and we found that DisA^K66R^ showed equivalent or similar intensities of cross-linking bands comparable to those obtained for DisA^WT^ (Fig. 1F). However, the DisA^K66Q^ mutant displayed a significantly reduced octamer form and an increased monomer form (Fig. 1F). These results revealed that the K66 residue was critical for DisA octamerization. The results of the DAC assay (Fig. 1G) led to the same conclusion that DisA^K66Q^ exhibited weak DAC activity. These results indicated that AcP-dependent acetylation at K66 dominated the impairment of DisA DAC activity.

**Fig 2:**
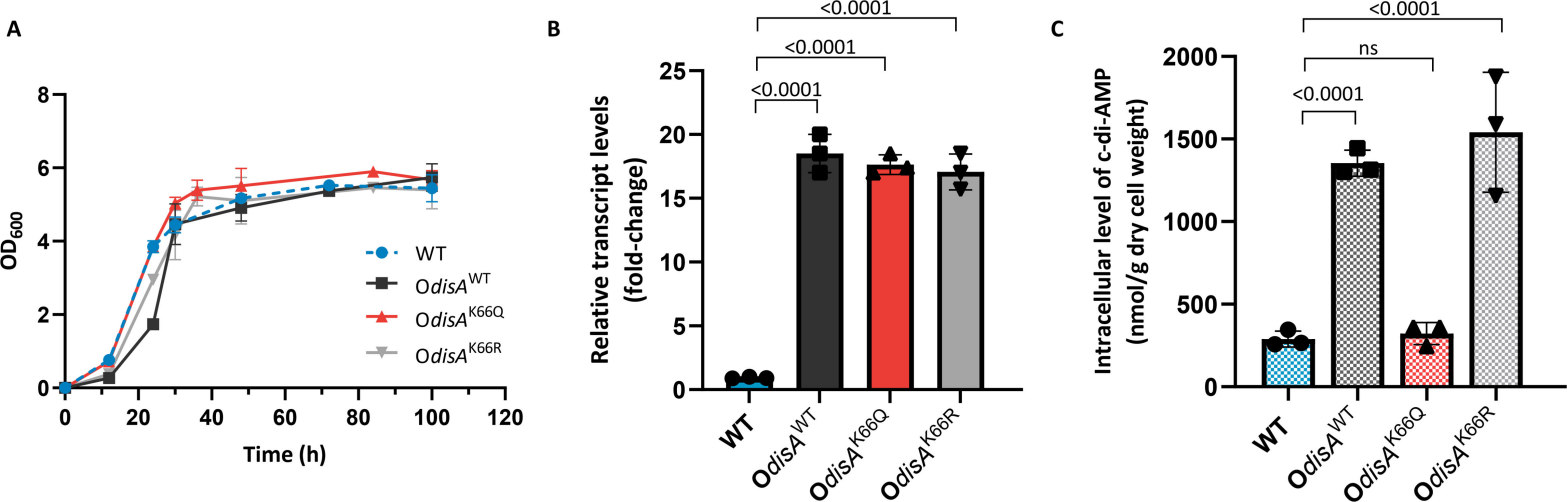
Effect of K66 acetylation on the synthesis of c-di-AMP *in vivo*. (**A**) Growth curves of the *S. erythraea* WT, O*disA*^WT^, O*disA*^K66Q^, and O*disA*^K66R^ strains grown at 30°C in trytone soy broth (TSB) media. (**B**) The *disA* transcription levels in the WT, O*disA*^WT^, O*disA*^K66Q^, and O*disA*^K66R^ strains grown in TSB media. The fold change represents the expression level compared to that of *disA* in the WT strain. (**C**) Intracellular c-di-AMP levels in cell extracts of the *S. erythraea* WT, O*disA*^WT^, O*disA*^K66Q^, and O*disA*^K66R^ strains grown in TSB media. The c-di-AMP concentrations of the samples were normalized to the dry cell weight. The error bars show the SDs of three independent experiments. Ordinary one-way analysis of variance was used for the statistical test.

Bacterial c-di-AMP synthesis is catalyzed by the DAC activity of DisA. To investigate whether K66 acetylation affects c-di-AMP synthesis in *S. erythraea*, we constructed DisA and its mutant overexpression strains as previously described (30). With the wild-type (WT) strain used as a control, we observed that both O*disA*^WT^ and O*disA*^K66R^ had relatively longer lag periods than the other strains (Fig. 2A); a possible reason might be that the overexpression of active DisA affects bacterial growth. The constructed strains were then analyzed by real-time RT-PCR, and the three DisA-overexpressing strains showed comparable *disA* transcription levels, which were more than a 15-fold greater than those of the WT strain (Fig. 2B). We next examined the effect of DisA and its mutant overexpression on intracellular c-di-AMP levels. A more than 75% drop in the intracellular c-di-AMP level of the O*disA*^K66Q^ strain was observed in comparison to that of the O*disA*^WT^ strain, and the O*disA*^K66R^ strain performed like the O*disA*^WT^ strain (Fig. 2C). In functional terms, these findings supported that DisA inactivation resulting from K66 acetylation directly abrogated its DAC activity and hence intracellular c-di-AMP synthesis directly.

### AcP-dependent acetylation eliminates the transcriptional control of *disA* via DasR deactivation

**Fig 3:**
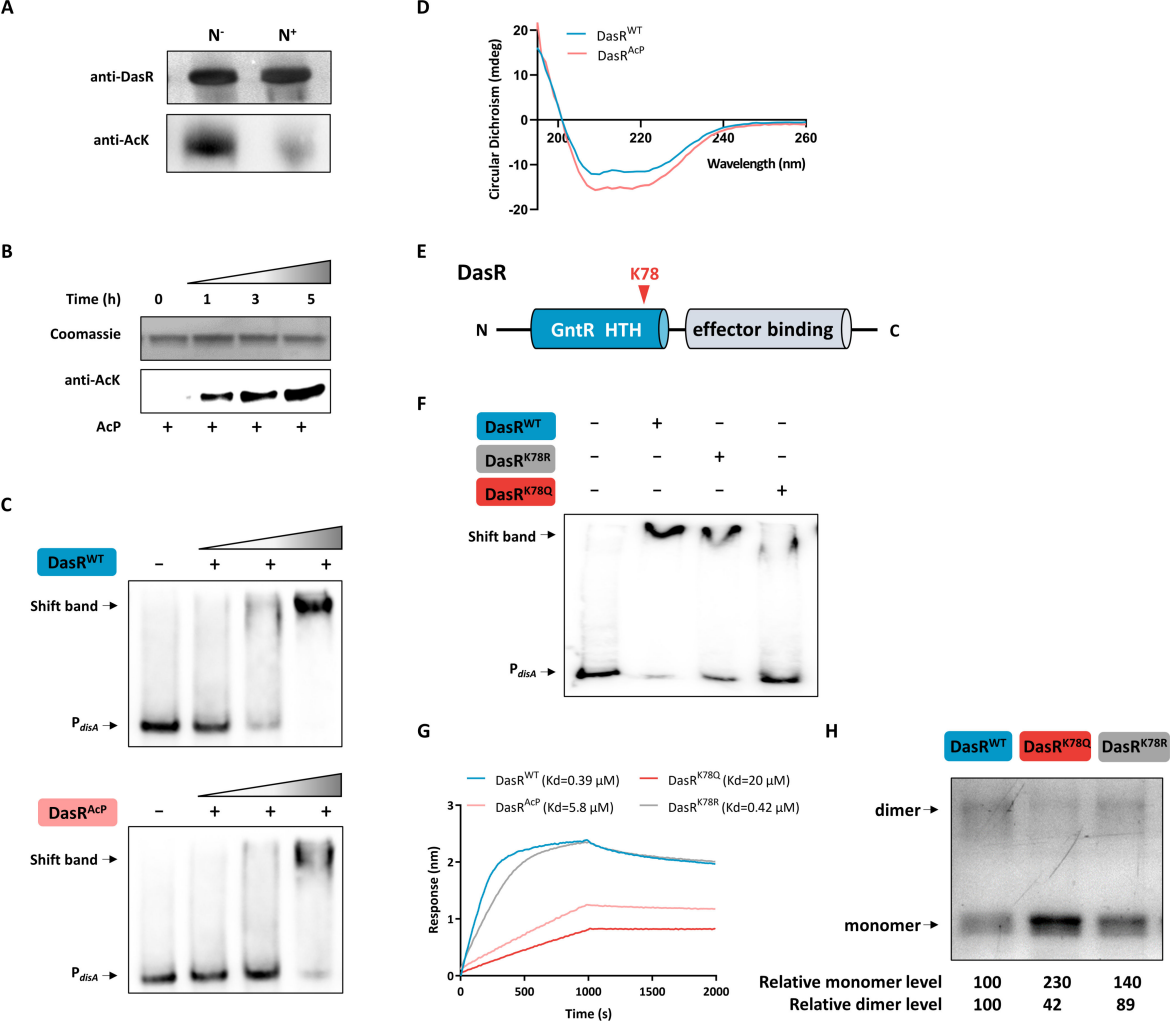
AcP-induced acetylation of DasR blocks its DNA-binding activity. (**A**) Acetylation level of DasR in the *S. erythraea* WT strain under N^+^ or N^−^ conditions. Each lane was loaded with an equal amount of DasR protein. (**B**) *In vitro* acetylation of His-tagged DasR protein with 10 mM AcP for various lengths of time (0, 1, 3, and 5 h) at 37°C. Acetylation levels were determined by Western blotting using a specific anti-AcK antibody. (**C**) EMSA of DasR^WT^ and DasR^AcP^ binding to its target gene *disA* promoter. (**D**) CD spectra of the DasR^WT^ and DasR^AcP^ proteins. (**E**) The domain organization of DasR is shown in differently colored boxes. The red inverted triangle shows the position of K78. (**F**) EMSA of DasR and its mutants binding to its target gene *disA* promoter. (**G**) Biolayer interferometry assay of purified His-DasR, DasR^AcP^, DasR^K78Q^, and DasR^K78R^ with the *disA* gene promoter. (**H**) Cross-linking of DasR and its mutants. The band intensities were quantified by densitometry using ImageJ software. The relative densitometry of the monomer and dimer in DasR^WT^ was set to 100.

Of note, *disA* was recently identified as a target gene of the global regulator DasR according to our latest study (31), in which DasR exerts direct transcriptional repression of c-di-AMP synthesis. We reasoned that AcP could also have an effect on the transcriptional control of *disA* expression via DasR acetylation. To test our hypothesis, the acetylation status of the DasR immunoprecipitates was determined with an anti-AcK antibody. As shown in Fig. 3A, DasR was hyperacetylated under nitrogen starvation conditions. *In vitro* acetylation further confirmed that DasR was acetylated by AcP (Fig. 3B). To investigate the effect of acetylation on the DNA-binding activity of the DasR regulator, electrophoretic mobility shift assay (EMSA) was performed using nonacetylated DasR (DasR^WT^) and AcP-acetylated DasR (DasR^AcP^). As shown in Fig. 3C, DasR^AcP^ showed weak DNA binding, as evidenced by the faint mobility shift. DasR belongs to the GntR-family regulators and interacts with DNA as a dimer (32). To determine whether AcP-induced acetylation influences DasR dimerization, a CD assay was performed. The Far-ultraviolet circular dichroism (Far-UV CD) spectra showed an increase in the α-helical content and a concomitant decrease in parallel and random coil structures compared with those of DasR^WT^, suggesting that acetylation altered the secondary structure of DasR (Fig. 3D; Table S1).

The proteomic data further showed that K78 was the only acetylation site identified in endogenous DasR (Table S2; Fig. S4 through 8). K78 is located in the GntR HTH domain, indicating a potential role of DNA binding (Fig. 3E). To verify this, we introduced substitutions at K78 to generate the DasR^K78Q^ and DasR^K78R^ variants. Together with DasR^WT^, the three proteins were subjected to EMSA, and we found that DasR^K78R^ performed similarly to DasR^WT^, while DasR^K78Q^ showed weak DNA binding, as evidenced by the slight mobility shift (Fig. 3F). To confirm this, we measured the Kd between DasR or its mutants and DNA using a biolayer interferometry (BLI) assay. As shown in Fig. 3G, DasR-DNA had a binding affinity of Kd ∼0.4 µM, whereas the Kd was ∼20 µM, a 50-fold increase for DasR^K78Q^-DNA, which was consistent with the data shown in Fig. 3E and F. Additionally, we found a reduced dimer form and an increased monomer form in DasR^K78Q^ (Fig. 3H), indicating that the dimerization function was also affected. These results reflected the inability of DasR to efficiently bind to the *disA* promoter after AcP-dependent acetylation at K78.

### DasR acetylation at K78 stimulates *disa* transcription and c-di-AMP synthesis

**Fig 4:**
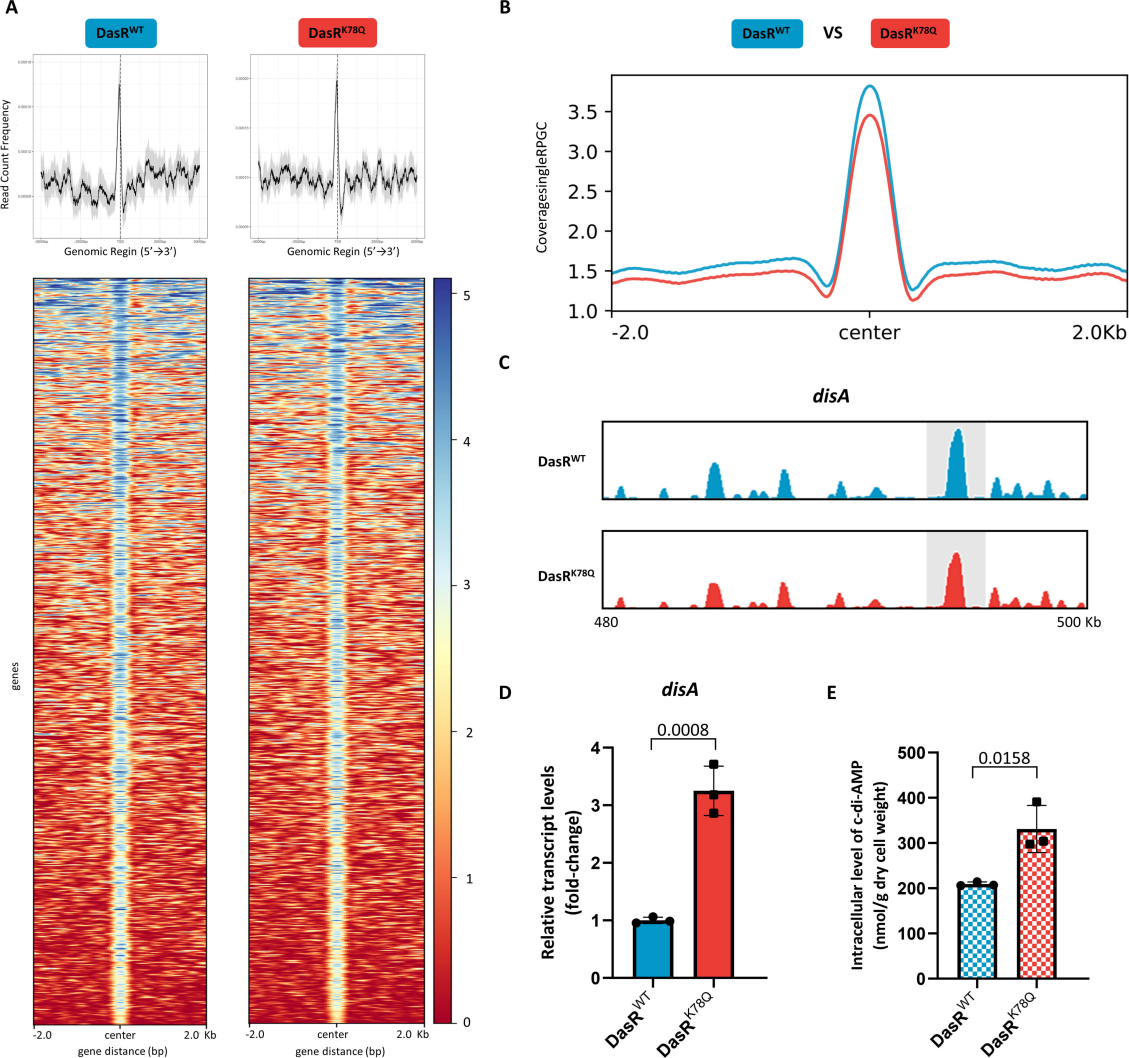
K78 acetylation releases the DasR-mediated repression of *disA in vivo*. (**A**) Heatmaps of the ChIP-seq signal density at the peak center and transcription start sites (±2 kb). The average signal profile is shown. Red indicates a low signal, and blue indicates a high signal. Source data are provided as a source data file. (**B**) ChIP-seq signal in the indicated strains. (**C**) Integrative Genomics Viewer tracks showing ChIP-seq signals at the promoter regions of *disA* in the indicated strains. (**D**) The transcription levels of *disA* in the indicated strains. The fold change represents the expression level compared to that of the DasR^WT^ strain. (**E**) Intracellular c-di-AMP levels in the indicated strains. The c-di-AMP concentrations of the samples were normalized to the dry cell weight. The error bars show the SDs of three independent experiments. A *t*-test was used for the statistical analysis.

To confirm and extend K78 acetylation of DasR in cells, we constructed *S. erythraea* strains overexpressing DasR^WT^ and DasR^K78Q^ using the *E. coli-S. erythraea* integrative shuttle vector pIB139 (33). The corresponding strains were subjected to chromatin immunoprecipitation sequencing (ChIP-seq) experiments using a specific anti-DasR antibody. The total input DNA (nonimmunoprecipitated) from each strain was also subjected to sequencing. Both the DasR^WT^ and DasR^K78Q^ signals were widely distributed at transcription start sites, with a sharp single peak (Fig. 4A). The total detected peaks showed downregulated binding signals in the DasR^K78Q^ strain compared with the DasR^WT^ strain (Fig. 4B). Consistently, representative results from the visualization and verification of *disA* during exposure to DasR^K78Q^ overexpression illustrated that the ChIP-seq peak changed at the individual gene level (Fig. 4C; Fig. S9). We therefore concluded that K78 acetylation attenuated the global outcomes of DasR binding to its targets, including *disA*. DasR, which predominantly functions as a global repressor, has a negative effect on *disA* expression (31). We then confirmed the ChIP-seq data by real-time RT-PCR to analyze the transcription of *disA*. As expected, the repression of *disA* was released in DasR^K78Q^ (Fig. 4D). In addition, the intracellular c-di-AMP concentration showed a consistent trend (Fig. 4E), supporting the idea that AcP signals through DasR to control the intracellular c-di-AMP level.

### c-di-AMP homeostasis mediates multicellular differentiation

**Fig 5:**
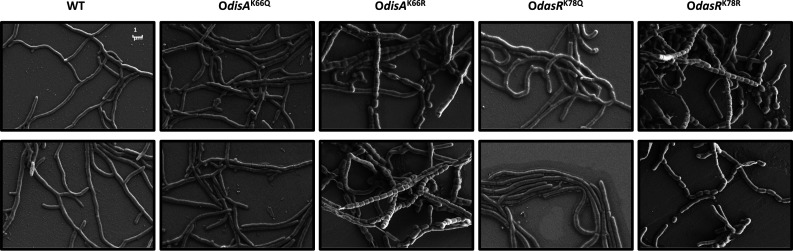
AcP-induced acetylation influences sporulation. Scanning electron micrographs of the *S. erythraea* WT, O*disA*^K66Q^, O*disA*^K66R^, O*dasR*^K78Q^, and O*dasR*^K78R^ strains grown for 96 h on R2YE agar at 30°C. The images are shown at ×5,000 magnification. Representative pictures of two independent experiments with similar results are shown.

The above two lines of evidence through transcriptional regulation and posttranslational regulation outline that AcP is a pivotal modulator of DisA, thereby providing a mechanism of c-di-AMP homeostasis control. First, DasR activation is hampered during acetylation at K78, indicating that this strain experiences excess c-di-AMP resulting from AcP-mediated indirect regulation. Second, DisA acetylation at K66 led to lower intracellular c-di-AMP amounts, indicating direct regulation caused by AcP-mediated acetylation. As cellular c-di-GMP levels were shown to control developmental transition in filamentous actinobacteria (20, 34, 35), we speculated that c-di-AMP might confer a similar function. To explore the possibility of this phenomenon being involved in developmental physiology and whether it is associated with AcP-mediated acetylation, the *S. erythraea* WT, O*disA*^K66Q^, O*disA*^K66R^, O*dasR*^K78Q^, and O*dasR*^K78R^ strains were cultivated onReversion 2 agar containing 0.5% yeast extract (R2YE) (36) at 30°C. The developmental phenotypes were examined by scanning electron microscopy (SEM), as shown in Fig. 5. The strains overexpressing DisA^K66Q^ showed a vegetative growth phenotype. In contrast, when DisA^K66R^ was overexpressed, we observed dramatic hypersporulation (Fig. 5), revealing that elevated c-di-AMP accumulation contributes to sporulation in *S. erythraea* and that K66 acetylation abolishes this facilitation. Compared with O*dasR*^K78R^, O*dasR*^K78Q^ showed delayed differentiation and sporulation (Fig. 5). These data indicated that c-di-AMP levels in response to acetylation status are critical to developmental fate.

### The physiological connection between AcP and c-di-AMP homeostasis is conserved

**Fig 6:**
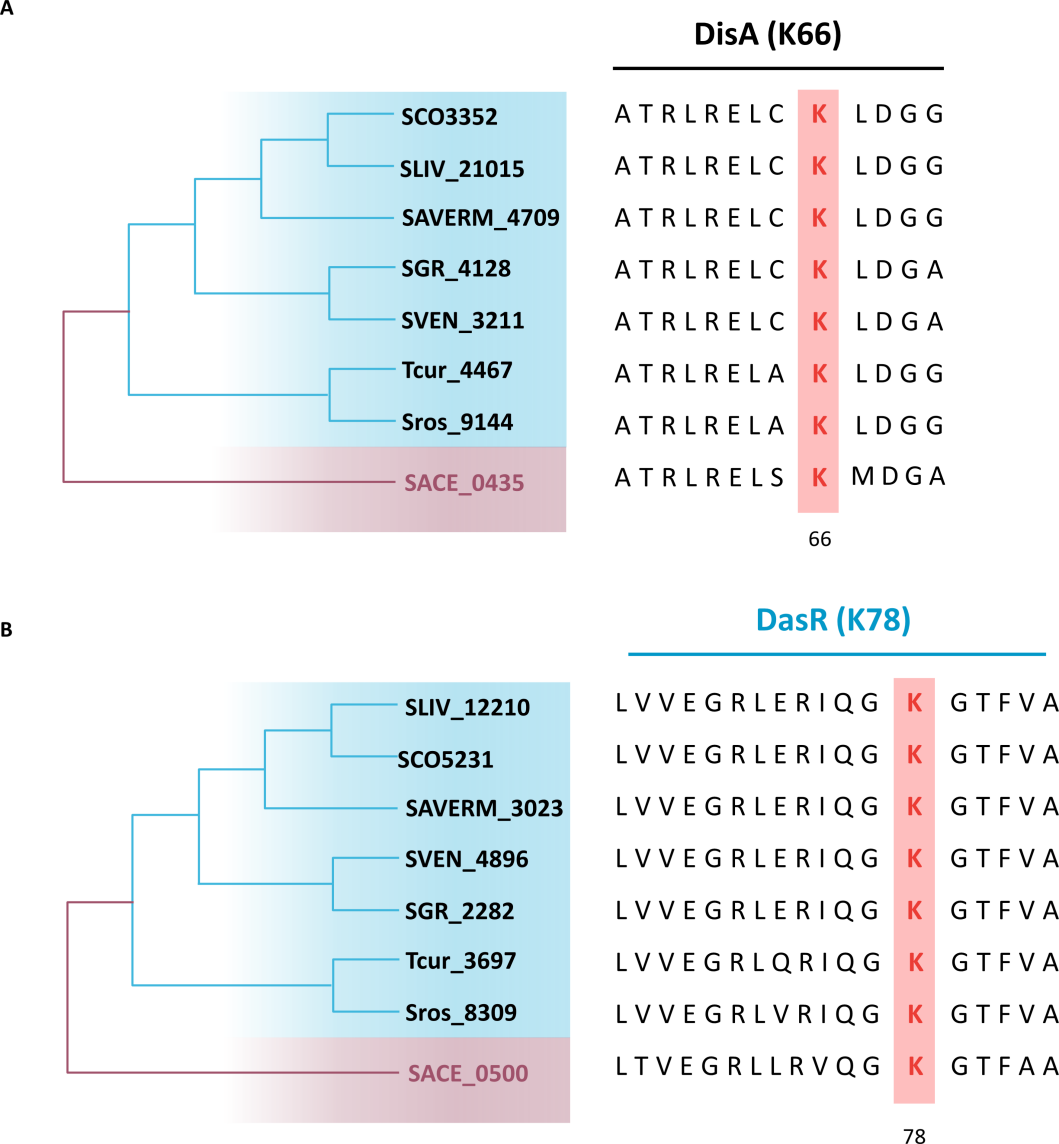
Sequence alignment of DasR and DisA proteins within actinobacteria. Alignments of the sequences of the *Streptomyces lividans*, *Streptomyces coelicolor*, *Streptomyces avermitilis*, *Streptomyces griseus*, *Streptomyces venezuelae*, *Streptosporangium roseum*, *Thermomonospora curvata*, and *S. erythraea* DisA (**A**) and DasR (**B**) proteins. The conserved lysine of all species is shown in bold red.

The fact that DisA_N domain-containing proteins (Pfam PF02457) are present in more than 11,000 bacterial and archaeal species raised the possibility that a similar regulation by AcP-induced acetylation might broadly exist. To complement these evolutionary insights, DisA homologs from *Streptomyces lividans*, *Streptomyces coelicolor*, *Streptomyces avermitilis*, *Streptomyces griseus*, *Streptomyces venezuelae, Streptosporangium roseum*, and *Thermomonospora curvata* were screened as representative actinobacteria for phylogenetic surveys. Indeed, according to the alignment of the *S. erythraea* DisA sequence with its homologs, K66 was highly conserved (Fig. 6A; Fig. S10). Intriguingly, we found the same results for DasR (Fig. 6B; Fig. S11). We predict, therefore, that a similar dependence on AcP contributes to c-di-AMP homeostasis and that changes in morphological differentiation may occur within actinobacteria subjected to constant nutrient stress.

## DISCUSSION

c-di-AMP governs numerous essential processes, including the osmotic state, biofilm formation, acid stress resistance, and the response to DNA damage and other functions, which have been studied extensively in Gram-negative bacteria (2). Variations in the c-di-AMP level (both high and low c-di-AMP levels) cause metabolic imbalance, which alters cell proliferation and related metabolic pathways (18). Mechanisms that ensure c-di-AMP maintenance are therefore crucial for cell function and have been the focus of intense scrutiny. Here, we identified a noncanonical route for DisA and hence c-di-AMP, which is modulated by direct or indirect regulation of AcP-dependent acetylation. Generally, DisA-like proteins are the most common group of DAC domain‐containing proteins in which the DAC domain is connected through a specific linker region to a DNA-binding domain. Such DisA proteins are mostly present in Gram‐positive spore-forming *Bacillus* and *Actinobacteria*. DisA forms a stable octamer with central pairs of DAC domains in solution, and the structure of the DisA octamer offers a plausible mechanism for the regulation of DAC activity (19). Indeed, our results indicated that DisA acetylation at K66 strongly inhibits diadenylate cyclase with a reduction in octamer formation. Such a conformational change in the protein could be the basis for the lower capability of four-way substrate binding. The intracellular c-di-AMP levels measured from cell extracts of *S. erythraea* WT strains under N^+^ or N^−^ conditions showed a decrease in the c-di-AMP concentration under N^−^ conditions compared to that under N^+^ conditions (Fig. S12), demonstrating that global hyperacetylation inhibited c-di-AMP accumulation.

**Fig 7:**
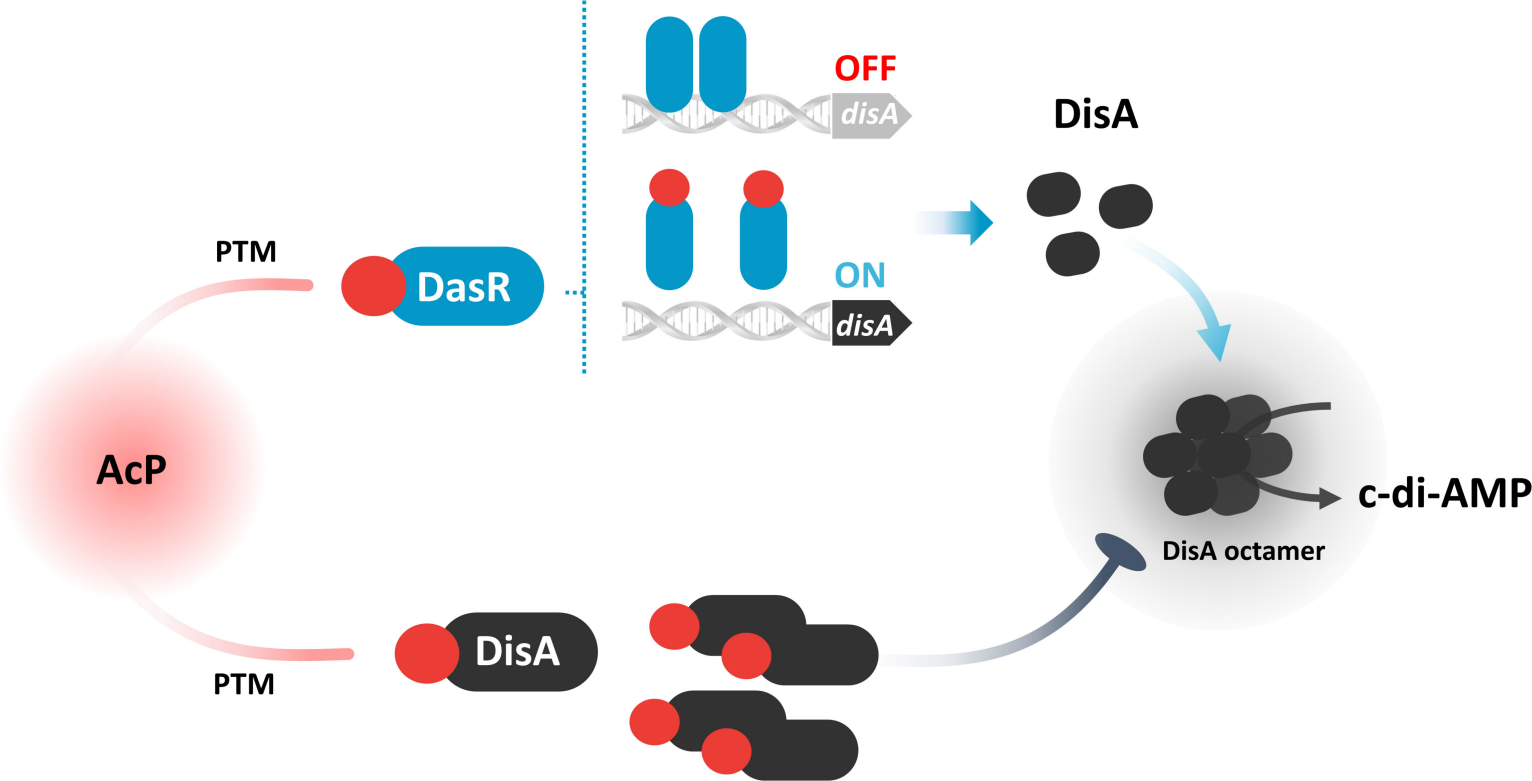
Schematic representation of the pathways involved in AcP control of c-di-AMP homeostasis. In the presence of AcP, the DasR residue K78 and the DisA residue K66 are acetylated (in red). DasR acetylation at K78 weakened its DNA binding and inhibited its transcriptional activity. Since DasR functions predominantly as a global repressor and has a negative effect on *disA* expression, the reduced DasR-DNA interaction caused by acetylation reversed the repression of *disA* and resulted in elevated *disA* transcription. DisA acetylation at K66 prevented its DAC activity, which inhibited c-di-AMP synthesis. PTM, posttranslational modification.

In addition to the posttranscriptional regulation of its enzymatic activity, the transcription of *disA* is under the direct transcriptional repression of the global pleiotropic regulator DasR, which is also regulated by AcP-related acetylation, to some extent resembling allosteric regulation. The network between AcP and DisA forms a homeostatic signal loop to maintain appropriate c-di-AMP states within the bacterial cell (Fig. 7), exhibiting therapeutic or industrial potential for preventing microbial growth. Intracellular survival within macrophages, which are thought to provide a nutritionally restricted environment, is important for mycobacterial pathogenesis. Although the mechanisms by which *M. tuberculosis* persists in macrophages remain largely unknown, intermediate metabolites, including AcP, acetyl-CoA, propionyl-CoA, and succinyl-CoA, are good targets for understanding the pathogenesis of mycobacteria. Importantly, it was suggested that, in addition to the primary metabolic defects, this metabolite-induced acylation modification could alter protein function and thus contribute to the pathophysiology. Notably, we found that AcP-dependent acetylation of DasR led to a loss of function in dimerization, which caused weakened DasR-DNA binding and inhibited its transcriptional activity, possibly exerting a global impact on actinobacterial physiology.

AcP-induced protein acetylation is generally considered a consequence of carbon overflow or an environmental carbon-nitrogen imbalance (24, 37, 38). Therefore, environmental cues can be integrated into a signaling network by affecting the activity of DisA, thereby controlling downstream phenotypic output. In all domains of life, nucleotide-based second messengers allow rapid integration of external and internal signals into regulatory pathways that control cellular responses to changing conditions. Previous studies have well explored that c-di-GMP acts as a brake on developmental transition through BldD and WhiG in the *Streptomyces* life cycle (20, 34). Based on our results, c-di-AMP has the opposite effect on developmental transition, which is under the fine-tuned regulation of AcP. It probably offers diverse and cooperative functions among different second messengers to recruit an appropriate signaling molecule and to decide between growth and sporulation. Due to the high conservation of the identified crucial sites (K66 of DisA and K78 of DasR) and the widespread AcP-triggered acetylation in bacteria, it is intriguing to speculate that the regulatory link between c-di-AMP homeostasis and the AcP signal in response to environmental stimuli is conserved in bacteria even though the specific molecular mechanisms may be completely different.

In summary, the identification of AcP-dependent acetylation established a network for DisA regulation at both the transcriptional and posttranslational levels, which contributes to global c-di-AMP homeostasis. Fundamental studies of c-di-AMP signaling have also revealed significant therapeutic promise. The decreased virulence associated with elevated c-di-AMP suggests that regulation of its production or hydrolysis may hold promise for therapeutic intervention in bacterial infections. Furthermore, the potent immunostimulatory activity of c-di-AMP and other cyclic dinucleotides shows promise as vaccine adjuvants for the prevention of infectious and malignant diseases. It also predicts that the regulatory network and the key acetylation sites described here may, in some instances, be a concept with important consequences for pathogen biology and new therapeutic discovery efforts.

## MATERIALS AND METHODS

### Bacterial strains and culture conditions

All strains and plasmids used in this study are listed in Table S3. The activation and preservation of all strains were described in detail in previous studies (39, 40). The *S. erythraea* wild-type strain (NRRL2338) and mutants were cultured in trytone soy broth or minimal Evans medium {25-mM N-[Tris(hydroxymethyl)methyl]-2-aminoethanesulfonic acid sodium salt, 10-mM KCl, 2-mM Na_2_SO_4_, 2-mM citric acid, 0.25-mM CaCl_2_, 1-µM Na_2_MoO_4_, 15-mM (NH_4_)_2_SO_4_, 2-mM KH_2_PO_4_, 2.5% (mass/volume) glucose, 0.5% trace elements (0.05-mM CoCl_2_·6H_2_O, 0.02-mM MnSO_4_·4H_2_O, 2-µM Na_2_MoO_4_·2H_2_O, 0.02-mM H_3_BO_3_, 1-µM KI, 6-µM ZnSO_4_·7H_2_O, and 0.05-mM CuSO_4_·5H_2_O}, pH 7.0, and supplemented with 1- or 15-mM (NH_4_)_2_SO_4_ for specific experiments.

### Overproduction and purification of proteins

The primers used for the amplification of *disA* and *dasR* from *S. erythraea* genomic DNA are listed in Table S4. The details of protein expression and purification were described in our previous works (39, 40). The purified proteins were analyzed by SDS-PAGE and the protein concentration was determined using BCA Protein Assay Kit [Tiangen Biotech (Beijing) Co., Ltd] with bovine serum albumin (BSA) as the standard.

### *In vitro* protein acetylation assays

*In vitro* acetylation assays with AcP were performed as previously described (41). Next, 10 µg of purified protein was added to a reaction mixture (total of 100 µL) containing 50-mM HEPES (pH 7.5) and 10-mM AcP. To investigate the effect of acetylation on the function of DisA/DasR, DisA/DasR and its mutants were incubated with or without AcP for 5 h. DisA^WT^/DasR^WT^ incubated without AcP for 5 h was used as a control, which could exclude the effects caused by the incubation periods. After incubation, the reaction samples were analyzed by Western blotting and LC-MS/MS.

### Identification of acetylated residues by LC-MS/MS

Protein samples were first separated by SDS-PAGE. The bands were then destained and dehydrated for further digestion and LC-MS/MS as described in detail in previous studies (42). The gel bands were sliced and destained in 50% ethanol. After being fully dehydrated in 100% acetonitrile (ACN), the samples were reduced by 10-mM dithiothreitol at 56°C for 40 min and then alkylated by 15-mM iodoacetamide in darkness for another 40 min. Then, the gels were washed in washing buffer [50% ACN/50% 50-mM NH_4_HCO_3_ (vol/vol)], and the proteins were digested by trypsin at an enzyme-to-substrate ratio of 1:40 for 16 h. The tryptic peptides were extracted in 50% ACN/5% trifluoroacetic acid (TFA), 75%/0.1% TFA, and 100% TFA in sequence. The samples were then dissolved in solvent A [0.1% (vol/vol) formic acid and 2% acetonitrile in water] and analyzed by an Orbitrap Fusion mass spectrometer in two technical replicates. The raw data were converted to mgf files and then analyzed by the Mascot search engine (v.2.3.01, Matrix Science). The search parameters were as follows: enzymes, trypsin/P; missed cleavage, 2; fixed modification, carbamidomethyl (C); variable modifications, acetylation (K), oxidation (M), and acetyl (protein N-terminal); peptide mass tolerance, 10 ppm; and fragment mass tolerance, 0.5 Da. The areas under the curve of the precursor ion peak were used to evaluate the peptide intensity. The ratios of the acylated peptides were normalized to the corresponding protein levels. Normalized ratios of the peptides were used for further analysis.

### Site-directed mutagenesis of acetylated site mutants

Using the primers listed in Table S4 and a fast mutagenesis system (TransGen Biotech, China), we introduced the mutants (K78Q and K78R) of DasR and the mutants (K66Q and K66R) of DisA into the pET28a (+) plasmid. After DNA sequencing confirmation, the recombinant plasmids were introduced into *E. coli* BL21 (DE3). The selected mutants were verified by PCR and DNA sequencing.

### IP and IB

Purification of the DisA and DasR proteins from *S. erythraea* through IP was performed as previously described (40) using specific anti-DisA and anti-DasR polyclonal antibodies (Solarbio, China), and the acetylation level was tested by IB using an anti‐AcK antibody (PTM-102, HangZhou Jingjie). The binding signal was visualized using an Omni-ECL Enhanced Pico Light Chemiluminescence Kit (Shanghai Epizyme Biomedical Technology Co., Ltd.) and scanned with a Tanon 4600 (Biotanon, China).

### Overexpression of DisA/DasR and its mutants in *S. erythraea*

The indicated strains were constructed as previously described (24). Briefly, overexpression plasmids were constructed by inserting the indicated sequence into the integrative shuttle vector pIB139 (33) between the NdeI and EcoRV restriction sites. This vector has been verified to have no effect on the bacterial physiology (43). The primers used are listed in Table S4. The recombinant plasmids were transformed into *S. erythraea* competent cells via polyethylene glycol and then integrated into the genome via phiC31. Apramycin resistance was used for selection. The selected mutants were confirmed by PCR and DNA sequencing.

### c-di-AMP quantification

As described previously by Corrigan et al. (44) with some modifications. Bacteria were collected by centrifugation (10,000 rpm, 30 min), washed, and freeze-dried after centrifugation to determine the dry weight for standardization purposes. The precipitate was suspended in 15–20 mL of ice-cold cell extraction buffer (ether:methanol:H_2_O, 40:40:20; liquid chromatography grade, VWR(Shanghai) Co.,Ltd, rapid freezing for 5 min). The samples were instantly frozen in liquid N_2_ for 10 min, heated to 95°C for 10 min, and held at 4°C for 30 min. Then, a cell crushing machine was used for ultrasonic lysis for 15 min. The supernatant was removed and stored at 4°C. The cell components were mixed and washed with extraction buffer, incubated on ice for 30 min and heated again. The sample was centrifuged again, and the supernatant was mixed with the previous supernatant. After freeze-drying, the samples were resuspended in 500 µL of cell extraction buffer for high-performance liquid chromatography (HPLC) (45) with a chromatographic analysis column RPC-18 (250 × 4.5 mm, Kromasil).

### DAC assays

DAC assays were performed as previously described (46). The reaction mixtures with purified 10-µM DisA protein (monomer concentration) in 0.1-M NaCl, 40-mM Tris (pH 7.5), and 10-mM MgCl_2_ were started by the addition of 100-mM ATP. At regular time intervals (every 15 min), the reactions were stopped with an equal volume of 0.5-M EDTA (pH 8.0). The samples were then analyzed for c-di-AMP production by HPLC. Enzyme activity was expressed as specific activity where unit of activity is the change in c-di-AMP production per minute per milligram of protein.

### EMSA

EMSAs were performed as previously described (30). DNA fragments and the upstream region from −300 to +50 of the detected genes were amplified by PCR using the primers listed in Table S4. The initial product was conjugated to 5′-biotin-AGCCAGTGGCGATAAG-3′ and purified with a HiPure PCR Pure Mini Kit (Magen, China). EMSAs were performed with a chemiluminescent EMSA kit (Beyotime Biotechnology, China) in a 10-µL reaction mixture containing 5-nM DNA and purified DasR. The DNA-protein mixture was incubated at 18°C for 30 min. Afterward, the samples were separated on a 6.5% polyacrylamide gel (Acr-Bis, 30%, 29:1) in 0.5 × Tris-borate-EDTA buffer at 380 mA. The bands were detected by an Omni-ECL Enhanced Pico Light Chemiluminescence Kit (Shanghai Epizyme Biomedical Technology Co., Ltd.) with a Tanon 4600 system (Biotanon).

### CD spectroscopy

CD spectroscopy was performed as previously described (47). The purified protein was replaced with CD buffer (1.4-M KF, 100-mM K_2_HPO_4_, and 18-mM KH_2_PO_4_, pH 7.4) and fixed to 0.2 mg/mL. Samples were measured at wavelengths of 180–260 nm at 25°C. For the analysis of the data, the background buffer signal was subtracted. After the processed data were exported, CDNN CD spectra deconvolution software (Applied Photophysics) was used for analysis of the secondary structure of the protein.

### BLI assay

A streptavidin (SA) biosensor purchased from ForteBio was used in this work. The loading buffer (pH 8.0) contained 10-mM HEPES, 2-mM MgCl_2_, 0.1-mM EDTA, and 200-mM KCl, and the running buffer contained an extra 10-µg/mL BSA and 0.02% Tween-20. The biotin-labeled DNA probe used was the same as that used for the EMSAs. The DNA probe was stored in loading buffer, and His-tagged DasR was stored in running buffer during the BLI assay with SA sensors. Samples were then detected within the OptiPlate-96 Black Opaque (PerkinElmer).

### Cross‐linking experiments

The protein was purified and dialyzed into cross-linked buffer [20-mM N,N-bis(2-hydroxyethyl)glycine (pH 8.0), 150-mM NaCl, 10-mM MgCl_2_, 0.2% n-octyl-β-D-glucopyranoside, and 8% glycerol]. The samples were treated with 1-mM disuccinimidyl suberate at room temperature for 1 h and then quenched with protein loading buffer. After heating to 95°C, the samples were separated on a 15% SDS polyacrylamide gel and visualized by Coomassie blue staining.

### ChIP-seq and data analysis

The DasR^WT^ and DasR^K78Q^ strains were subsequently grown for the appropriate length of time. Formaldehyde was added to the cultures at a final concentration of 1% (vol/vol), and the incubation was continued for 30 min. Glycine was then added to a final concentration of 125 mM to stop the cross-linking. The samples were left at room temperature for 10 min and washed twice in precooled TBS buffer (pH 7.5) containing 20-mM Tris-HCl and 150-mM NaCl. ChIP-seq was performed by E-GENE Tech Co., Ltd. (Shenzhen) using an anti-DasR polyclonal antibody. Briefly, fastp software (v.0.20.0) was used to trim adaptors and remove low-quality reads to obtain high-quality clean reads. Clean reads were aligned to the reference genome using Bowtie2 software (v.2.2.4). MACS2 software (v.2.2.7.1) was used for peak calling. Bedtools software (v.2.30.0) was used for peak annotation based on GTF annotation files. Homer (v.4.11) software was used to identify motifs. MAnorm2 (v.1.2.0) software was used to identify differentially enriched regions. The enriched peaks were visualized with Integrative Genomics Viewer (v.2.4.10) software.

### Western blotting

Western blotting was performed as previously described (40). Protein lysates were separated by 10% SDS-PAGE and then transferred to PVDF membranes for 60 min at 100 V. After blocking with 3% BSA in phosphate-buffered saline containing 0.1% Tween-80 buffer at room temperature for 1 h, an anti‐AcK antibody (PTM-102, HangZhou Jingjie) diluted 1:15,000 in Tris-buffered saline with tween (TBST)/5% BSA was used. The blot was performed via an ImageQuant LAS 4000 system (GE Healthcare, UK) after chemiluminescent Horseradish Peroxidase (HRP) substrate treatment.

### RNA preparation and real-time RT-PCR

Total RNA extraction was performed according to our previous work (48). Total RNA (1 µg) was used to synthesize cDNA using the PrimeScript RT Reagent Kit with gDNA Eraser (Takara). 16S rRNA (SACE_8101) was used as the internal standard. The resulting cDNA was diluted and used as a template for real-time RT-PCR with SYBR Premix Ex Taq GC (Takara). The primers used are listed in Table S4. PCR assays were performed on a CFX96 real-time system (Bio-Rad, Hercules, CA) with the following PCR conditions: 95°C for 5 min, 40 cycles at 95°C for 5 s and at 60°C–64°C for 30 s, and an extension at 72°C for 10 min. The transcriptional variations were analyzed by the threshold cycle (2^−∆∆CT^) method.

### Scanning electron microscopy

*S. erythraea* strains were cultivated on R2YE agar plates (36) covered with plastic cellophane at 30°C. At the specified times, a piece of cellophane covered with mycelia was extracted, fixed with 2% osmium tetroxide for 24 h, and then dehydrated by air drying for 1 h. Each specimen was sputter coated with platinum gold and examined with a Gemini SEM 500 scanning electron microscope.

## Data Availability

Chromatin immunoprecipitation sequencing raw data are available in the GEO database (accession ID: GSE239753). All study data are included in the article and/or supplemental material.

## SUPPLEMENTAL MATERIAL

Supplemental Material mbio.01411-24-s0001.docx Supplemental tables and figures.

Source Data File mbio.01411-24-s0002.svg The source data for Fig. 4A.

ASM does not own the copyrights to Supplemental Material that may be linked to, or accessed through, an article. The authors have granted ASM a non-exclusive, world-wide license to publish the Supplemental Material files. Please contact the corresponding author directly for reuse.

## References

[1] Cereija TB, Guerra JPL, Jorge JMP, Morais-Cabral JH. 2021. C-Di-AMP, a likely master regulator of bacterial K(+) homeostasis machinery, activates a K(+) exporter. Proc Natl Acad Sci U S A 118:e2020653118. doi:10.1073/pnas.2020653118 33790011 PMC8040634

[2] He J, Yin W, Galperin MY, Chou SH. 2020. Cyclic Di-AMP, a second messenger of primary importance: tertiary structures and binding mechanisms. Nucleic Acids Res 48:2807–2829. doi:10.1093/nar/gkaa112 32095817 PMC7102992

[3] Stülke J, Krüger L. 2020. Cyclic Di-AMP signaling in bacteria. Annu Rev Microbiol 74:159–179. doi:10.1146/annurev-micro-020518-115943 32603625

[4] Yoon SH, Waters CM. 2021. The ever-expanding world of bacterial cyclic oligonucleotide second messengers. Curr Opin Microbiol 60:96–103. doi:10.1016/j.mib.2021.01.017 33640793 PMC8026173

[5] Sureka K, Choi PH, Precit M, Delince M, Pensinger DA, Huynh TN, Jurado AR, Goo YA, Sadilek M, Iavarone AT, Sauer JD, Tong L, Woodward JJ. 2014. The cyclic dinucleotide c-di-AMP is an allosteric regulator of metabolic enzyme function. Cell 158:1389–1401. doi:10.1016/j.cell.2014.07.046 25215494 PMC4166403

[6] Kim H, Youn SJ, Kim SO, Ko J, Lee JO, Choi BS. 2015. Structural studies of potassium transport protein KtrA regulator of conductance of K+ (RCK) C domain in complex with cyclic diadenosine monophosphate (c-di-AMP). J Biol Chem 290:16393–16402. doi:10.1074/jbc.M115.641340 25957408 PMC4481236

[7] Choi PH, Sureka K, Woodward JJ, Tong L. 2015. Molecular basis for the recognition of cyclic-di-AMP by PstA, a PII-like signal transduction protein. Microbiologyopen 4:361–374. doi:10.1002/mbo3.243 25693966 PMC4475381

[8] Gundlach J, Herzberg C, Kaever V, Gunka K, Hoffmann T, Weiß M, Gibhardt J, Thürmer A, Hertel D, Daniel R, Bremer E, Commichau FM, Stülke J. 2017. Control of potassium homeostasis is an essential function of the second messenger cyclic Di-AMP in Bacillus subtilis Sci Signal 10:eaal3011. doi:10.1126/scisignal.aal3011 28420751

[9] Gundlach J, Krüger L, Herzberg C, Turdiev A, Poehlein A, Tascón I, Weiss M, Hertel D, Daniel R, Hänelt I, Lee VT, Stülke J. 2019. Sustained sensing in potassium homeostasis: cyclic di-AMP controls potassium uptake by KimA at the levels of expression and activity. J Biol Chem 294:9605–9614. doi:10.1074/jbc.RA119.008774 31061098 PMC6579464

[10] Yin W, Cai X, Ma H, Zhu L, Zhang Y, Chou SH, Galperin MY, He J. 2020. A decade of research on the second messenger c-di-AMP. FEMS Microbiol Rev 44:701–724. doi:10.1093/femsre/fuaa019 32472931 PMC7850090

[11] Huynh TN, Choi PH, Sureka K, Ledvina HE, Campillo J, Tong L, Woodward JJ. 2016. Cyclic di-AMP targets the cystathionine beta-synthase domain of the osmolyte transporter OpuC. Mol Microbiol 102:233–243. doi:10.1111/mmi.13456 27378384 PMC5118871

[12] Moscoso JA, Schramke H, Zhang Y, Tosi T, Dehbi A, Jung K, Gründling A. 2016. Binding of cyclic di-AMP to the Staphylococcus aureus sensor kinase KdpD occurs via the universal stress protein domain and downregulates the expression of the Kdp potassium transporter. J Bacteriol 198:98–110. doi:10.1128/JB.00480-15 26195599 PMC4686210

[13] Moscoso JA, Schramke H, Zhang Y, Tosi T, Dehbi A, Jung K, Gründling A. 2015. Binding of c-di-AMP to the Staphylococcus aureus sensor kinase KdpD occurs via the USP domain and down-regulates the expression of the Kdp potassium transporter. J Bacteriol 198:98–110. doi:10.1128/JB.00480-15 26195599 PMC4686210

[14] Whiteley AT, Garelis NE, Peterson BN, Choi PH, Tong L, Woodward JJ, Portnoy DA. 2017. c-di-AMP modulates listeria monocytogenes central metabolism to regulate growth, antibiotic resistance and osmoregulation. Mol Microbiol 104:212–233. doi:10.1111/mmi.13622 28097715 PMC5391996

[15] Woodward JJ, Iavarone AT, Portnoy DA. 2010. C-Di-AMP secreted by intracellular listeria monocytogenes activates a host type I interferon response. Science 328:1703–1705. doi:10.1126/science.1189801 20508090 PMC3156580

[16] Devaux L, Kaminski PA, Trieu-Cuot P, Firon A. 2018. Cyclic di-AMP in host-pathogen interactions. Curr Opin Microbiol 41:21–28. doi:10.1016/j.mib.2017.11.007 29169058

[17] Zhang L, Li W, He ZG. 2013. DarR, a TetR-like transcriptional factor, is a cyclic di-AMP-responsive repressor in Mycobacterium smegmatis. J Biol Chem 288:3085–3096. doi:10.1074/jbc.M112.428110 23250743 PMC3561532

[18] Huynh TN, Woodward JJ. 2016. Too much of a good thing: regulated depletion of c-di-AMP in the bacterial cytoplasm. Curr Opin Microbiol 30:22–29. doi:10.1016/j.mib.2015.12.007 26773214 PMC4821758

[19] Witte G, Hartung S, Büttner K, Hopfner K-P. 2008. Structural biochemistry of a bacterial checkpoint protein reveals diadenylate cyclase activity regulated by DNA recombination intermediates. Mol Cell 30:167–178. doi:10.1016/j.molcel.2008.02.020 18439896

[20] Bush MJ, Tschowri N, Schlimpert S, Flärdh K, Buttner MJ. 2015. c-di-GMP signalling and the regulation of developmental transitions in streptomycetes. Nat Rev Microbiol 13:749–760. doi:10.1038/nrmicro3546 26499894

[21] Liu G, Chater KF, Chandra G, Niu G, Tan H. 2013. Molecular regulation of antibiotic biosynthesis in streptomyces. Microbiol Mol Biol Rev 77:112–143. doi:10.1128/MMBR.00054-12 23471619 PMC3591988

[22] van Wezel GP, McDowall KJ. 2011. The regulation of the secondary metabolism of streptomyces: new links and experimental advances. Nat Prod Rep 28:1311–1333. doi:10.1039/c1np00003a 21611665

[23] Hopwood DA. 1999. Forty years of genetics with streptomyces: from in vivo through in vitro to in Silico. Microbiology (Reading) 145 ( Pt 9):2183–2202. doi:10.1099/00221287-145-9-2183 10517572

[24] Fu Y, Dong YQ, Shen JL, Yin BC, Ye BC, You D. 2023. A meet-up of acetyl phosphate and c-di-GMP modulates BldD activity for development and antibiotic production. Nucleic Acids Res 51:6870–6882. doi:10.1093/nar/gkad494 37283056 PMC10359459

[25] Peng ZY, Fu Y, Zhao LC, Dong YQ, Chen ZQ, You D, Ye BC. 2023. Protein acylation links metabolism and the control of signal transduction, transcription regulation, growth, and pathogenicity in actinobacteria. Mol Microbiol 119:151–160. doi:10.1111/mmi.14998 36349384

[26] Walsh CT, Garneau‐Tsodikova S, Gatto GJ Jr. 2005. Protein posttranslational modifications: the chemistry of proteome diversifications. Angew Chem 44:7342–7372. doi:10.1002/anie.200501023 16267872

[27] Christensen DG, Baumgartner JT, Xie X, Jew KM, Basisty N, Schilling B, Kuhn ML, Wolfe AJ. 2019. Mechanisms, detection, and relevance of protein acetylation in prokaryotes. mBio 10:e02708–02718. doi:10.1128/mBio.02708-18 30967470 PMC6456759

[28] VanDrisse CM, Escalante-Semerena JC. 2019. Protein acetylation in bacteria. Annu Rev Microbiol 73:111–132. doi:10.1146/annurev-micro-020518-115526 31091420 PMC6736716

[29] Ren Q, Gorovsky MA. 2001. Histone H2A.Z acetylation modulates an essential charge patch. Mol Cell 7:1329–1335. doi:10.1016/s1097-2765(01)00269-6 11430834

[30] You D, Yin BC, Li ZH, Zhou Y, Yu WB, Zuo P, Ye BC. 2016. Sirtuin-dependent reversible lysine acetylation of glutamine synthetases reveals an autofeedback loop in nitrogen metabolism. Proc Natl Acad Sci U S A 113:6653–6658. doi:10.1073/pnas.1525654113 27247389 PMC4914186

[31] You D, Zhao LC, Fu Y, Peng ZY, Chen ZQ, Ye BC. 2024. Allosteric regulation by c-di-AMP modulates a complete N-acetylglucosamine signaling cascade in Saccharopolyspora erythraea. Nat Commun 15:3825. doi:10.1038/s41467-024-48063-0 38714645 PMC11076491

[32] Jain D. 2015. Allosteric control of transcription in GntR family of transcription regulators: a structural overview. IUBMB Life 67:556–563. doi:10.1002/iub.1401 26172911

[33] Wilkinson CJ, Hughes-Thomas ZA, Martin CJ, Böhm I, Mironenko T, Deacon M, Wheatcroft M, Wirtz G, Staunton J, Leadlay PF. 2002. Increasing the efficiency of heterologous promoters in actinomycetes. J Mol Microbiol Biotechnol 4:417–426. 12125822

[34] Tschowri N, Schumacher MA, Schlimpert S, Chinnam NB, Findlay KC, Brennan RG, Buttner MJ. 2014. Tetrameric c-di-GMP mediates effective transcription factor dimerization to control streptomyces development. Cell 158:1136–1147. doi:10.1016/j.cell.2014.07.022 25171413 PMC4151990

[35] Gallagher KA, Schumacher MA, Bush MJ, Bibb MJ, Chandra G, Holmes NA, Zeng W, Henderson M, Zhang H, Findlay KC, Brennan RG, Buttner MJ. 2020. c-di-GMP arms an anti-sigma to control progression of multicellular differentiation in streptomyces. Mol Cell 77:586–599. doi:10.1016/j.molcel.2019.11.006 31810759 PMC7005675

[36] Thompson CJ, Ward JM, Hopwood DA. 1980. DNA cloning in streptomyces: resistance genes from antibiotic-producing species. Nature 286:525–527. doi:10.1038/286525a0 6250070

[37] Schilling B, Basisty N, Christensen DG, Sorensen D, Orr JS, Wolfe AJ, Rao CV. 2019. Global lysine acetylation in Escherichia coli results from growth conditions that favor acetate fermentation. J Bacteriol 201:e00768-18. doi:10.1128/JB.00768-18 30782634 PMC6456854

[38] Weinert BT, Iesmantavicius V, Wagner SA, Schölz C, Gummesson B, Beli P, Nyström T, Choudhary C. 2013. Acetyl-phosphate is a critical determinant of lysine acetylation in E. coli. Mol Cell 51:265–272. doi:10.1016/j.molcel.2013.06.003 23830618

[39] You D, Wang MM, Ye BC. 2017. Acetyl-CoA synthetases of Saccharopolyspora erythraea are regulated by the nitrogen response regulator GlnR at both transcriptional and post-translational levels. Mol Microbiol 103:845–859. doi:10.1111/mmi.13595 27987242

[40] You D, Yao LL, Huang D, Escalante-Semerena JC, Ye BC. 2014. Acetyl coenzyme a synthetase is acetylated on multiple lysine residues by a protein acetyltransferase with a single Gcn5-type N-acetyltransferase (GNAT) domain in Saccharopolyspora erythraea. J Bacteriol 196:3169–3178. doi:10.1128/JB.01961-14 24957627 PMC4135648

[41] You D, Wang M-M, Ye B-C. 2017. Acetyl-CoA synthetases of Saccharopolyspora erythraeaare regulated by the nitrogen response regulator GlnR at both transcriptional and post-translational levels. Mol Microbiol 103:845–859. doi:10.1111/mmi.13595 27987242

[42] Xu J-Y, Xu Y, Xu Z, Zhai L-H, Ye Y, Zhao Y, Chu X, Tan M, Ye B-C. 2018. Protein acylation is a general regulatory mechanism in biosynthetic pathway of Acyl-CoA-derived natural products. Cell Chem Biol 25:984–995. doi:10.1016/j.chembiol.2018.05.005 29887264

[43] Xu Z, You D, Tang LY, Zhou Y, Ye BC. 2019. Metabolic engineering strategies based on secondary messengers (P)ppGpp and c-di-GMP to increase erythromycin yield in Saccharopolyspora erythraea*.* ACS Synth Biol 8:332–345. doi:10.1021/acssynbio.8b00372 30632732

[44] Corrigan RM, Abbott JC, Burhenne H, Kaever V, Gründling A. 2011. c-di-AMP is a new second messenger in Staphylococcus aureus with a role in controlling cell size and envelope stress. PLoS Pathog 7:e1002217. doi:10.1371/journal.ppat.1002217 21909268 PMC3164647

[45] Oppenheimer-Shaanan Y, Wexselblatt E, Katzhendler J, Yavin E, Ben-Yehuda S. 2011. c-di-AMP reports DNA integrity during sporulation in Bacillus subtilis. EMBO Rep 12:594–601. doi:10.1038/embor.2011.77 21566650 PMC3128283

[46] Chan C, Paul R, Samoray D, Amiot NC, Giese B, Jenal U, Schirmer T. 2004. Structural basis of activity and allosteric control of diguanylate cyclase. Proc Natl Acad Sci U S A 101:17084–17089. doi:10.1073/pnas.0406134101 15569936 PMC535365

[47] Zhang BQ, Chen ZQ, Dong YQ, You D, Zhou Y, Ye BC. 2022. Selective recruitment of stress-responsive mRNAs to ribosomes for translation by acetylated protein S1 during nutrient stress in Escherichia coli. Commun Biol 5:892. doi:10.1038/s42003-022-03853-4 36050442 PMC9437053

[48] You D, Zhang B-Q, Ye B-C. 2018. GntR family regulator DasR controls acetate assimilation by directly repressing the acsA gene in Saccharopolyspora erythraea. J Bacteriol 200:e00685-17. doi:10.1128/JB.00685-17 29686136 PMC5996686

