# Supplementary material for: A network of acetyl phosphate-dependent modification modulates c-di-AMP homeostasis in Actinobacteria
Source: mBio. 2024 Jul 9;15(8):e01411-24. doi: 10.1128/mbio.01411-24 (PMC11323494; doi:10.1128/mbio.01411-24)
Supplement: Supplemental Material — Supplemental tables and figures. [file mbio.01411-24-s0001.docx]

Supplementary Material:

**A network of acetyl phosphate-dependent modification modulates c-di-AMP homeostasis in *Actinobacteria***

Yu Fu^1#^, Liu-Chang Zhao^1#^, Jin-Long Shen^1^, Shi-Yu Zhou^1^, Bin-Cheng Yin^1^, Bang-Ce Ye^1,2*^, Di You^1*^

^1^Laboratory of Biosystems and Microanalysis, State Key Laboratory of Bioreactor Engineering, East China University of Science and Technology, Shanghai 200237, China;

^2^Institute of Engineering Biology and Health, Collaborative Innovation Center of Yangtze River Delta Region Green Pharmaceuticals, College of Pharmaceutical Sciences, Zhejiang University of Technology, Hangzhou 310014, Zhejiang, China.

^#^ Yu Fu and Liu-Chang Zhao contributed equally to this work

^*^ Corresponding author

**Corresponding authors**

Bang-Ce Ye

Professor, Lab of Biosystems and Microanalysis,

State Key Laboratory of Bioreactor Engineering,

East China University of Science and Technology, Shanghai 200237, China

Tel/Fax: 0086-21-64252094

Email: [bcye@ecust.edu.cn](mailto:bcye@ecust.edu.cn)

Di You

Associate Professor, Lab of Biosystems and Microanalysis,

State Key Laboratory of Bioreactor Engineering,

East China University of Science and Technology, Shanghai 200237, China

Tel/Fax: 0086-21-64253832

Email: [030111115@mail.ecust.edu.cn](mailto:030111115@mail.ecust.edu.cn)

**Table S1 Secondary structural elements in DisA and DasR**

| **Protein** | **Helix**  **（%）** | **Antiparallel**  **（%）** | **Parallel**  **（%）** | **β-turn**  **（%）** | **Random coil**  **（%）** | **Total sum**  **（%）** |
| --- | --- | --- | --- | --- | --- | --- |
| **DisA^WT^** | 99.9 | 0 | 0 | 1.9 | 0.1 | 101.9 |
| **DisA^AcP^** | 99.7 | 0.1 | 0.1 | 3.2 | 0.3 | 103.2 |
| **DasR^WT^** | 92.2 | 0 | 0.9 | 7.6 | 2.2 | 102.9 |
| **DasR^AcP^** | 95.3 | 0 | 0.6 | 6.5 | 1.2 | 103.5 |

**Table S2 The acetylated sites of DisA and DasR**

| **Protein** | **Acetylation sites** | |
| --- | --- | --- |
|  | ***In vitro*** | ***In vivo*** |
| **DisA** | **K66, K284** | **K66** |
| **DasR** | **K21，K78，K84，K86** | **K78** |

**Table S3 Strains and plasmids used in this work**

| **Strain or plasmid** | **Characteristics** | **Source or Reference** |
| --- | --- | --- |
| **Strains** |  |  |
| *E. coli* DH5α | *E. coli* DH5α F‐Ø80d lacZΔM (lacZYA ‐argF) U169 deoR | Transgen Biotech |
| *E. coli*BL21(DE3) | F'ompTr‐ Bm‐B (DE3) | Transgen Biotech |
| *E. coli*BL21(DE3)- *dasR*^WT^ | The strain for expression of DasR^WT^ | This study |
| *E. coli*BL21(DE3)- *dasR*^K78Q^ | The strain for expression of DasR^K78Q^ | This study |
| *E. coli*BL21(DE3)- *dasR*^K78R^ | The strain for expression of DasR^K78R^ | This study |
| *E. coli*BL21(DE3)- *disA*^WT^ | The strain for expression of DisA^WT^ | This study |
| *E. coli*BL21(DE3)- *disA*^K66Q^ | The strain for expression of DisA^K66Q^ | This study |
| *E. coli*BL21(DE3)- *disA*^K66R^ | The strain for expression of DisA^K66R^ | This study |
| *S. erythraea* NRRL2338 | Used as parental strain, wild type | DSM 40517 |
| *S. erythraea*O*dasR*^WT^ | The strain for over‐expression of *dasR*, NRRL2338 integrated with pIB139‐*dasR* | This study |
| *S. erythraea*O*dasR*^K78R^ | The strain for over‐expression of *dasR*^K78R^, NRRL2338 integrated with pIB139‐*dasR*^K78R^ | This study |
| *S. erythraea*O*dasR*^K78Q^ | The strain for over‐expression of *dasR*^K78Q^, NRRL2338 integrated with pIB139‐*dasR*^K78Q^ | This study |
| *S. erythraea OdisA*^WT^ | The strain for over‐expression of *disA*^WT^ NRRL2338 integrated with pIB139‐*disA*^WT^ | This study |
| *S. erythraea OdisA*^K66Q^ | The strain for over‐expression of *disA*^K66Q^ NRRL2338 integrated with pIB139‐*disA*^K66Q^ | This study |
| *S. erythraea OdisA*^K66R^ | The strain for over‐expression of *disA*^K66R^ NRRL2338 integrated with pIB139‐*disA*^K66R^ | This study |
| **plasmid** |  |  |
| pET28a(+) | vector with T7‐RNA polymerase‐  based promoter for expression in  *E. coli* BL21(DE3), hexahistidine  tag with thrombin cleavage | Thermo Scientific |
| pET- *dasR*^WT^ | pET28a(+) with *dasR*^WT^ | This study |
| pET- *dasR*^K78Q^ | pET28a(+) with *dasR*^K78Q^ | This study |
| pET- *dasR*^K78R^ | pET28a(+) with *dasR*^K78R^ | This study |
| pET- *disA*^WT^ | pET28a(+) with *disA*^WT^ | This study |
| pET- *disA*^K66Q^ | pET28a(+) with *disA*^K66Q^ | This study |
| pET- *disA*^K66R^ | pET28a(+) with *disA*^K66R^ | This study |
| pIB139 | pSET152 with integrase of phiC31 and PermE, the strong promoter of *Streptomyces* |  |
| pIB139- *dasR*^WT^ | pIB139 with *dasR*^WT^ gene | This study |
| pIB139- *dasR*^K78Q^ | pIB139 with *dasR*^K78Q^ gene | This study |
| pIB139- *dasR*^K78R^ | pIB139 with *dasR*^K78R^ gene | This study |
| pIB139- *disA*^WT^ | pIB139 with *disA*^WT^ gene | This study |
| pIB139- *disA*^K66Q^ | pIB139 with *disA*^K66Q^ gene | This study |
| pIB139- *disA*^K66R^ | pIB139 with *disA*^K66R^ gene | This study |

**Table S4 The oligonucleotides used in the study**

| Oligonucleotides | Sequence (5’to 3’) |
| --- | --- |
| **Primers for overproduction of DasR and DisA proteins** | |
| pET-*dasR*F | AGCAAATGGGTCGCGGATCCCTCGAAACATCGGTGCCAAGC |
| pET-*dasR*R | CGAGTGCGGCCGCAAGCTTGGGCGGGCGGGTTGAGG |
| pET-*disA*F | atgggcagcagccatcatcatcatcatcacgtgaacgagaagctgcgcgccac |
| pET-*disA*R | cggatctcagtggtggtggtggtggtgtcaggcgtagcggtccatgatcgagg |
| T7F | TAATACGACTCACTATAGGGGAATTGTGAGCG |
| T7R | CAAAAAACCCCTCAAGACCCGTTTAGAGG |
| **Primers for the construction of the *S. erythraea* O*dasR* and O*disA* strains** | |
| pIB-*dasR*F | aatgggtcgcggatccgaattcatgctcgaaacatcggtgccaagc |
| pIB-*dasR*R | gctcgagtgcggccgcaagctttcaggcgggcgggttgagg |
| pIB-*disA*F | aatgggtcgcggatccgaattcgtgaacgagaagctgcgcgccacg |
| pIB-*disAR* | gctcgagtgcggccgcaagctttcaggcgtagcggtccatgatcgagg |
| M13F | cgccagggttttcccagtcacgac |
| M13R | cacacaggaaacagctatgac |
| **The primers used in site-directed mutagenesis of DasR** | |
| K78R F | ctgcgcgtccagggcaGgggcacctt |
| K78R R | gccctggacgcgcagcagcctgccct |
| K78Q F | ctgcgcgtccagggcCAgggcacctt |
| K78Q R | gccctggacgcgcagcagcctgccct |
| **The primers used in site-directed mutagenesis of DisA** | |
| K66R F | gctgcgcgagctgtccaGgatggacg |
| K66R R | ggacagctcgcgcagccgggtcgcg |
| K66Q F | gctgcgcgagctgtcccagatggacg |
| K66Q R | ggacagctcgcgcagccgggtcgcg |
| **Primers for PCR amplification of EMSAs probe with biotin labeling** | |
| *disA* | AGCCAGTGGCGATAAGCGGCCTGACCCAGGACATCACCAC |
|  | AGCCAGTGGCGATAAGCGGGCTCACCCACCCGTCGGCATGT |
| **Primers for real-time RT-PCR** | |
| SACE_8101-F | CAAAGGAATTGACGGGGGC |
| SACE_8101-R | CACGGGGTCGAGTTGCAGA |
| *disA*-F | ACGGTGCGGTGGTGCTC |
| *disA*-R | CCGACAGGGTCTGGGCG |

**
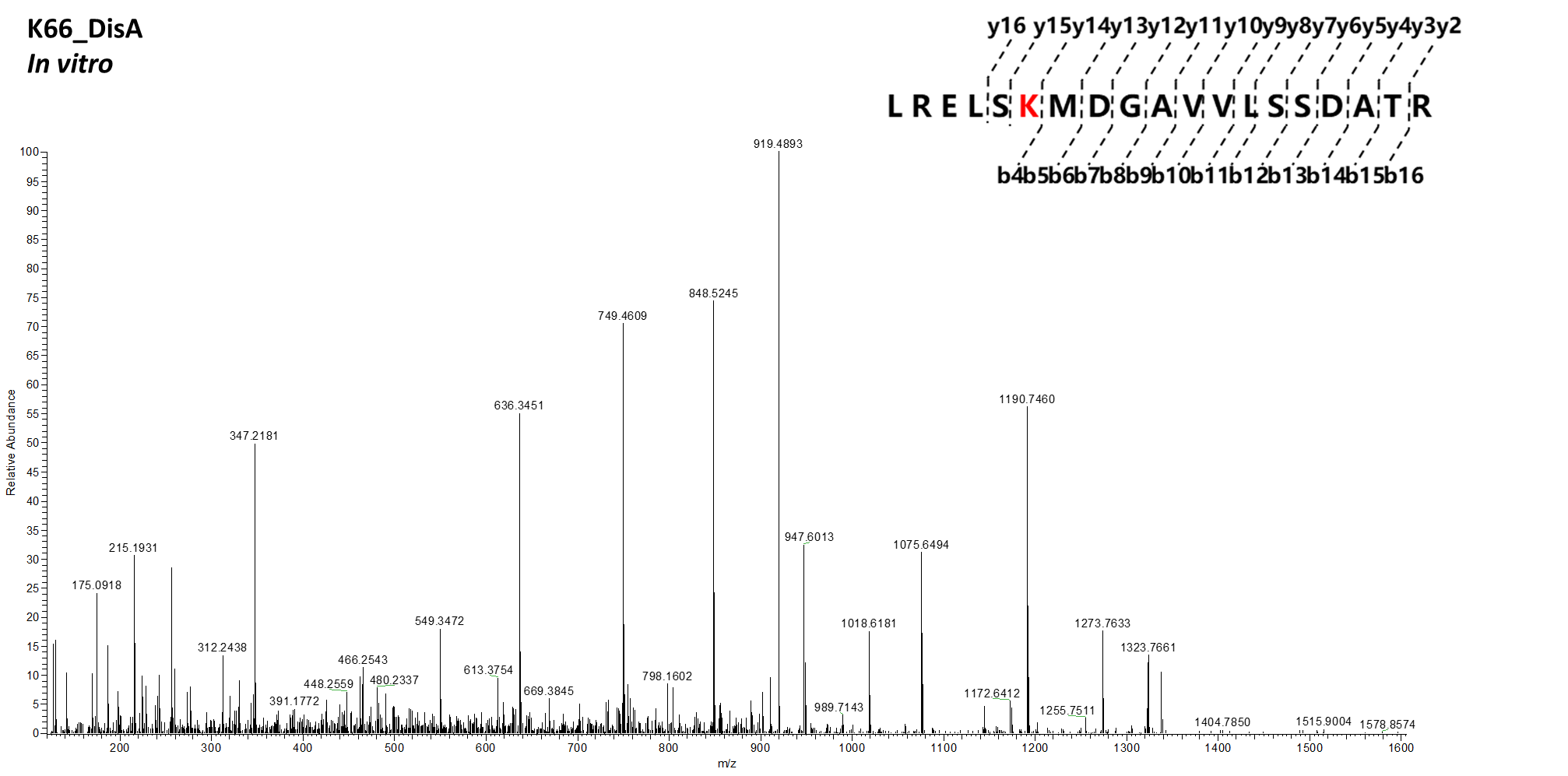
**

**Figure S1** **MS/MS spectra for the identification of K66 *in vitro* by LC-MS/MS analysis.**

**
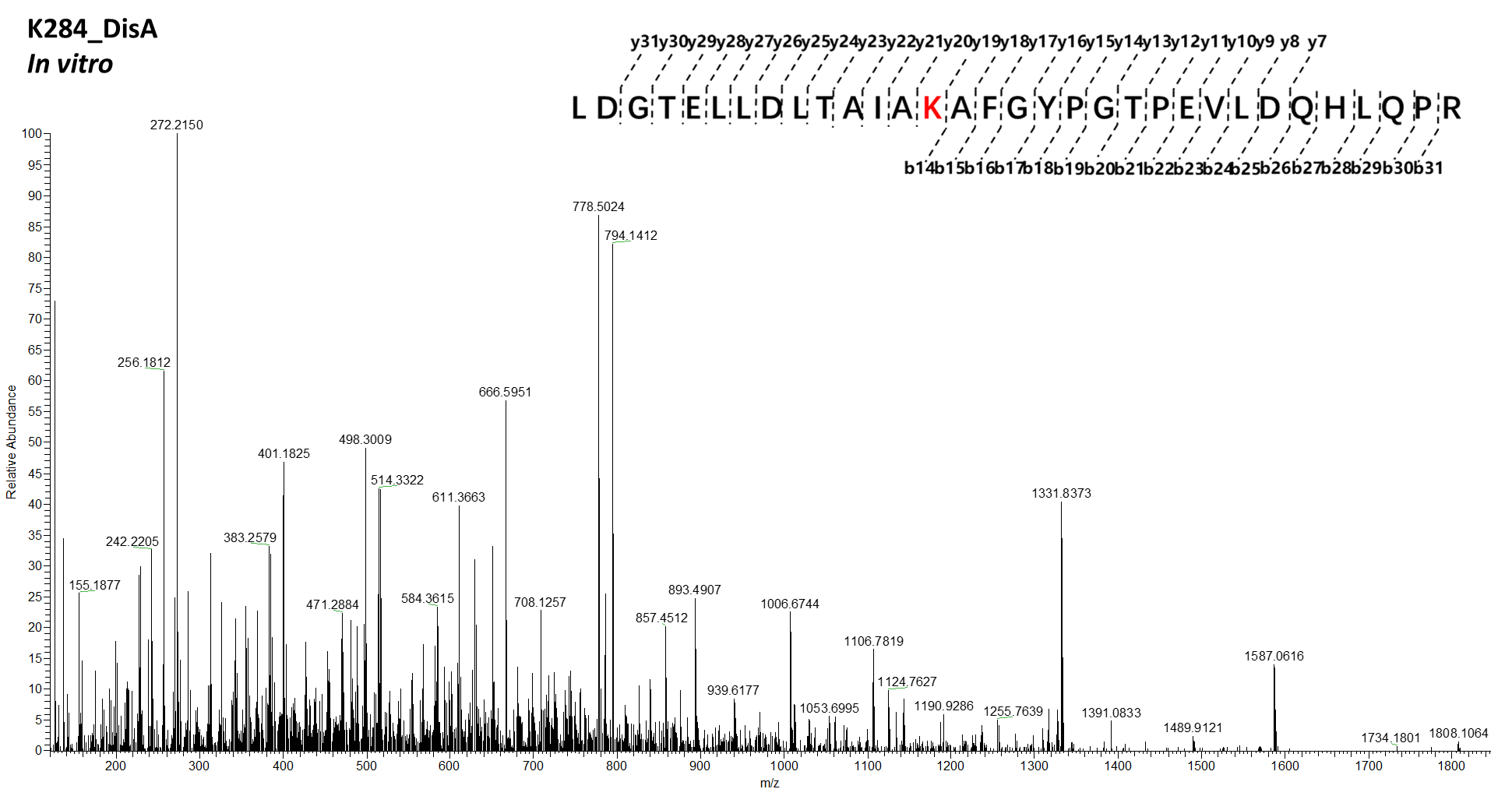
**

**Figure S2** **MS/MS spectra for the identification of K284 *in vitro* by LC-MS/MS analysis.**

**
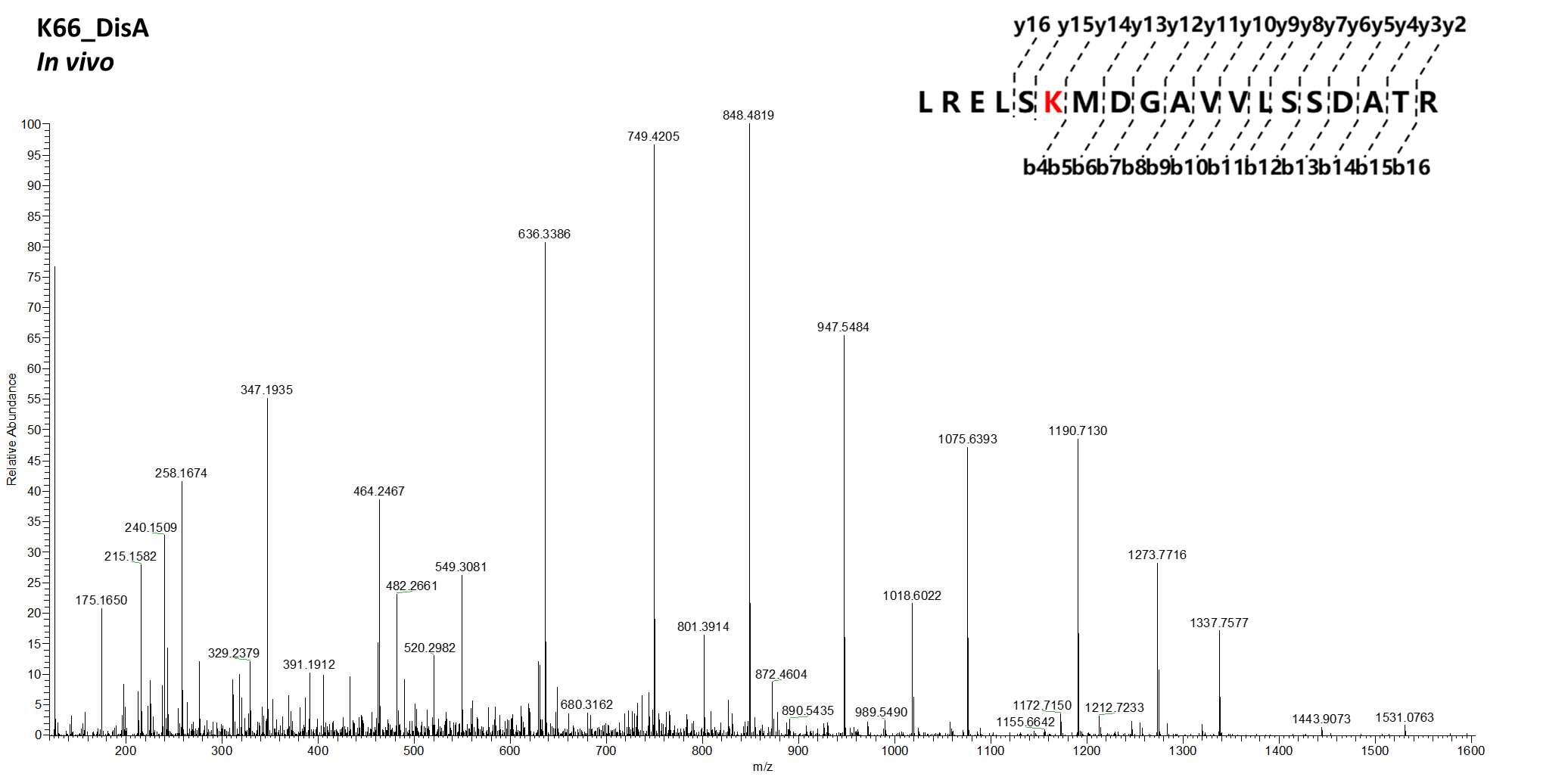
**

**Figure S3** **MS/MS spectra for the identification of K66 *in vivo* by LC-MS/MS analysis.**

**
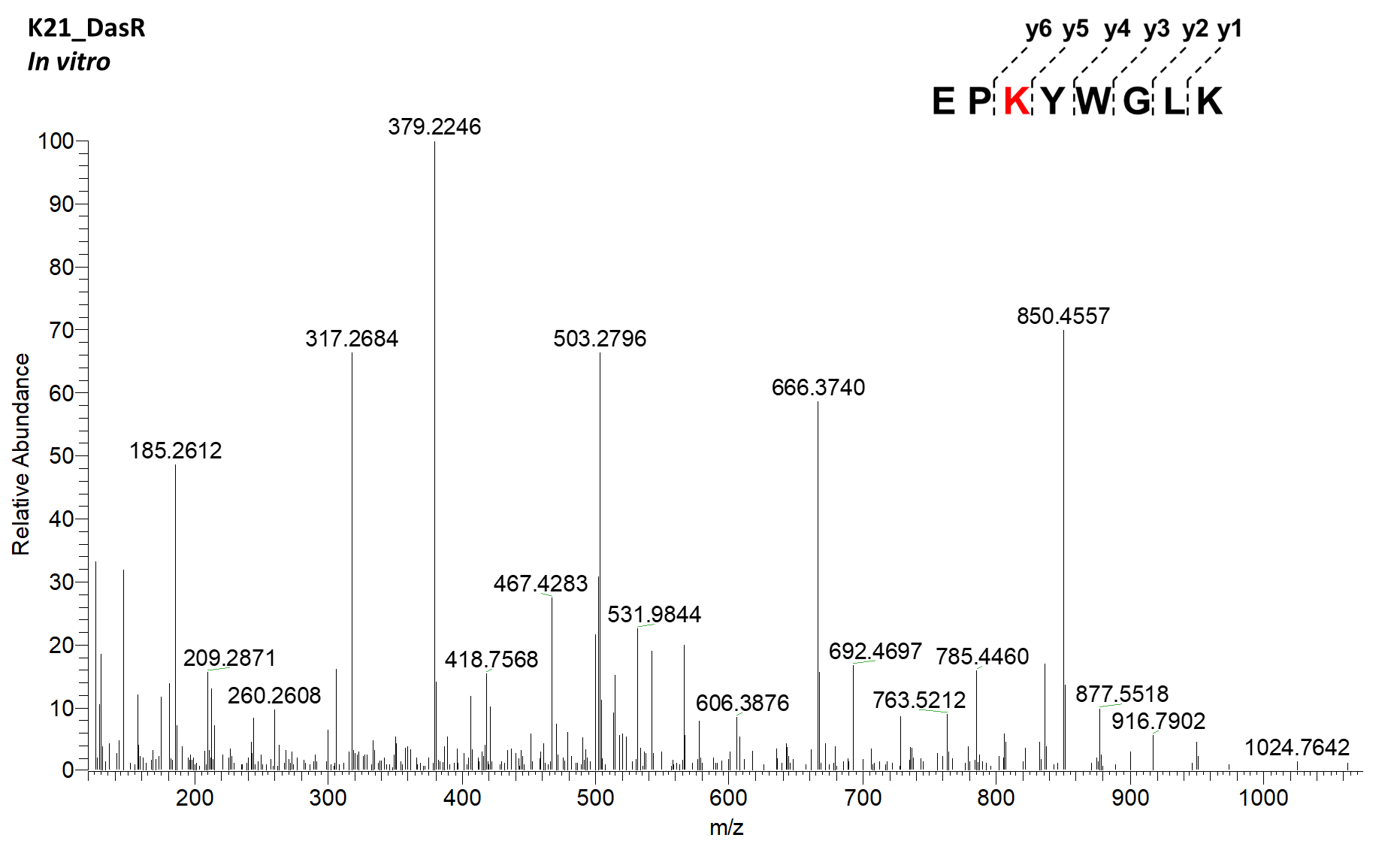
**

**Figure S4** **MS/MS spectra for the identification of K21 *in vitro* by LC-MS/MS analysis.**

**
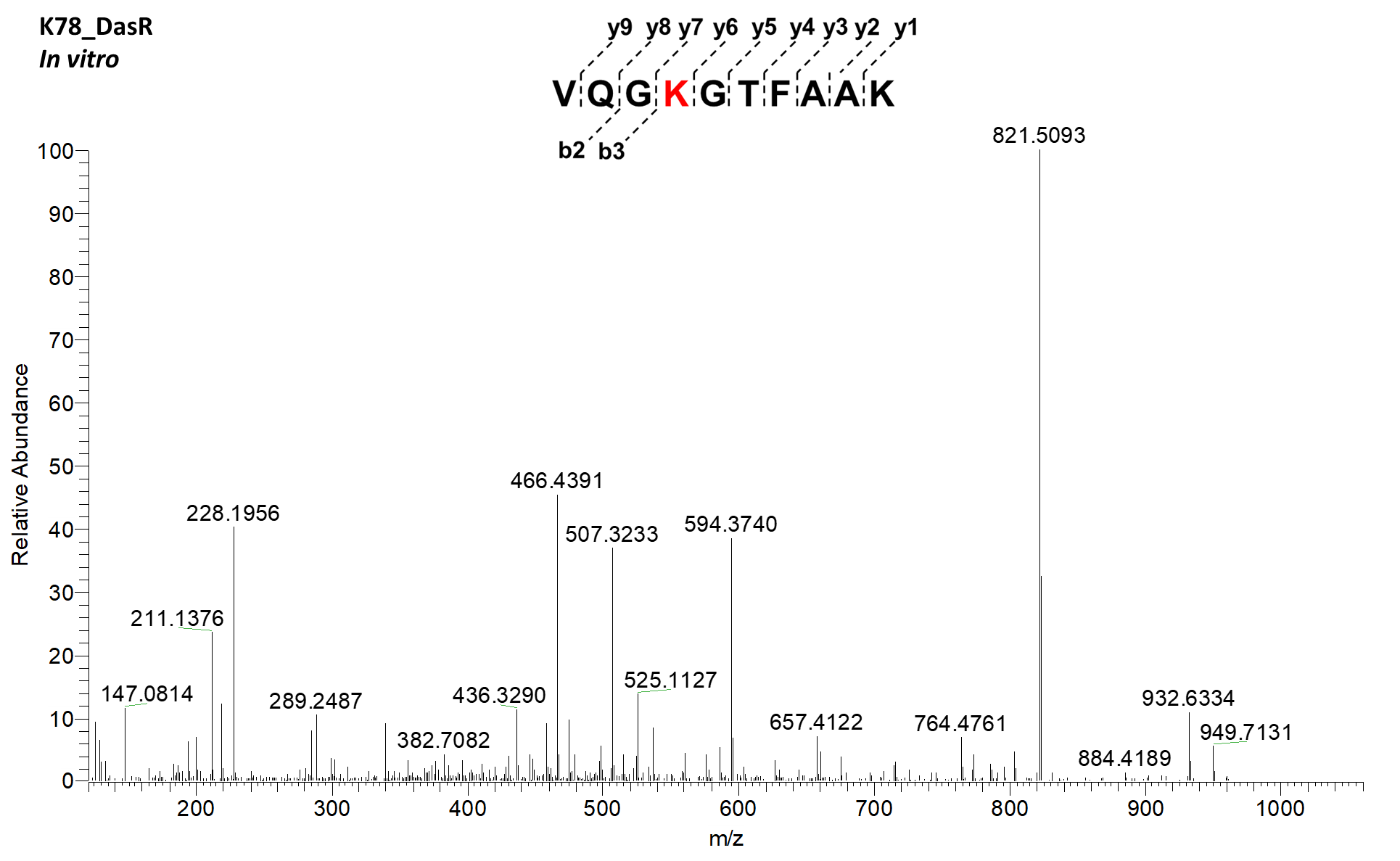
**

**Figure S5** **MS/MS spectra for the identification of K78 *in vitro* by LC-MS/MS analysis.**

**
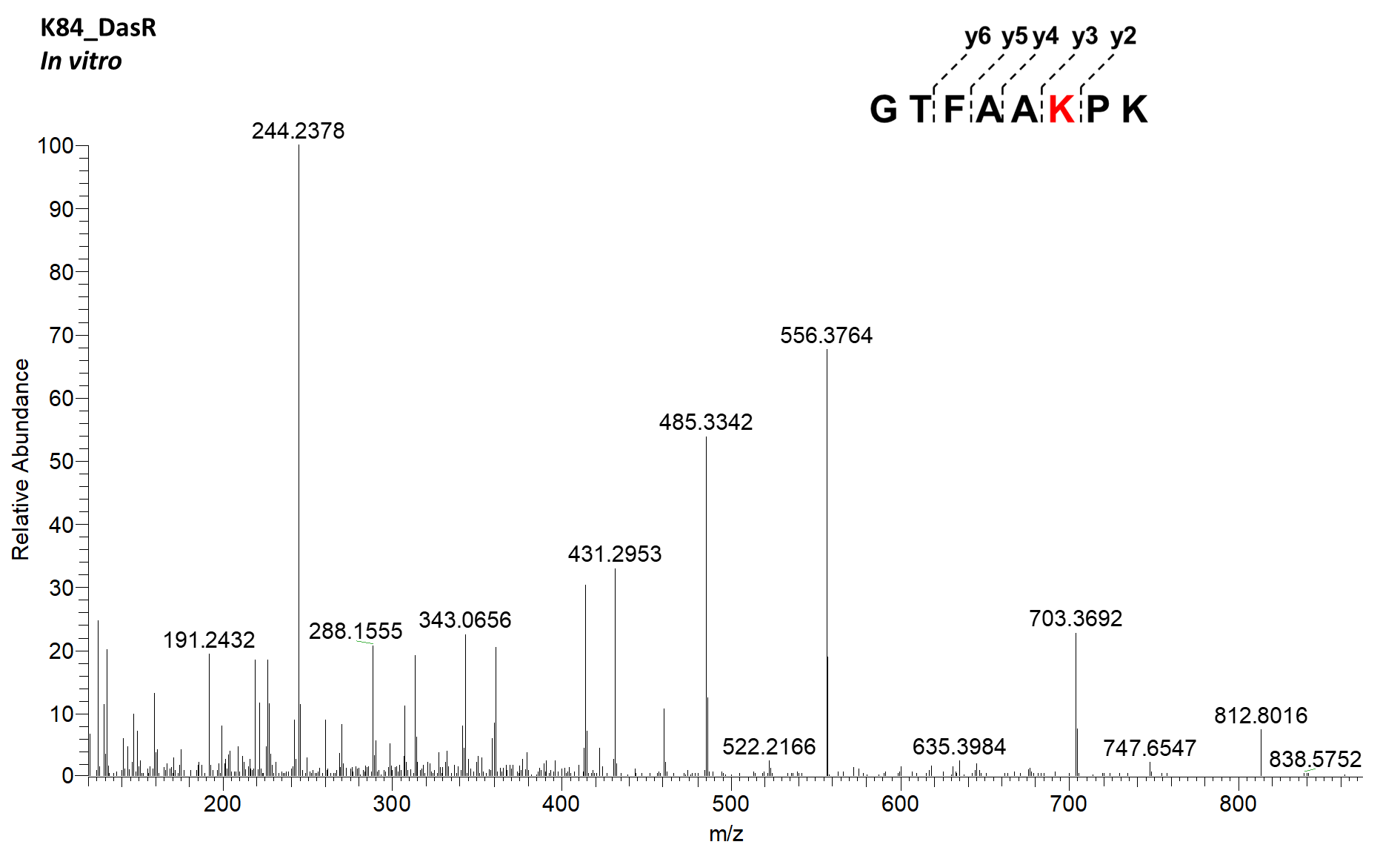
**

**Figure S6** **MS/MS spectra for the identification of K84 *in vitro* by LC-MS/MS analysis.**

**
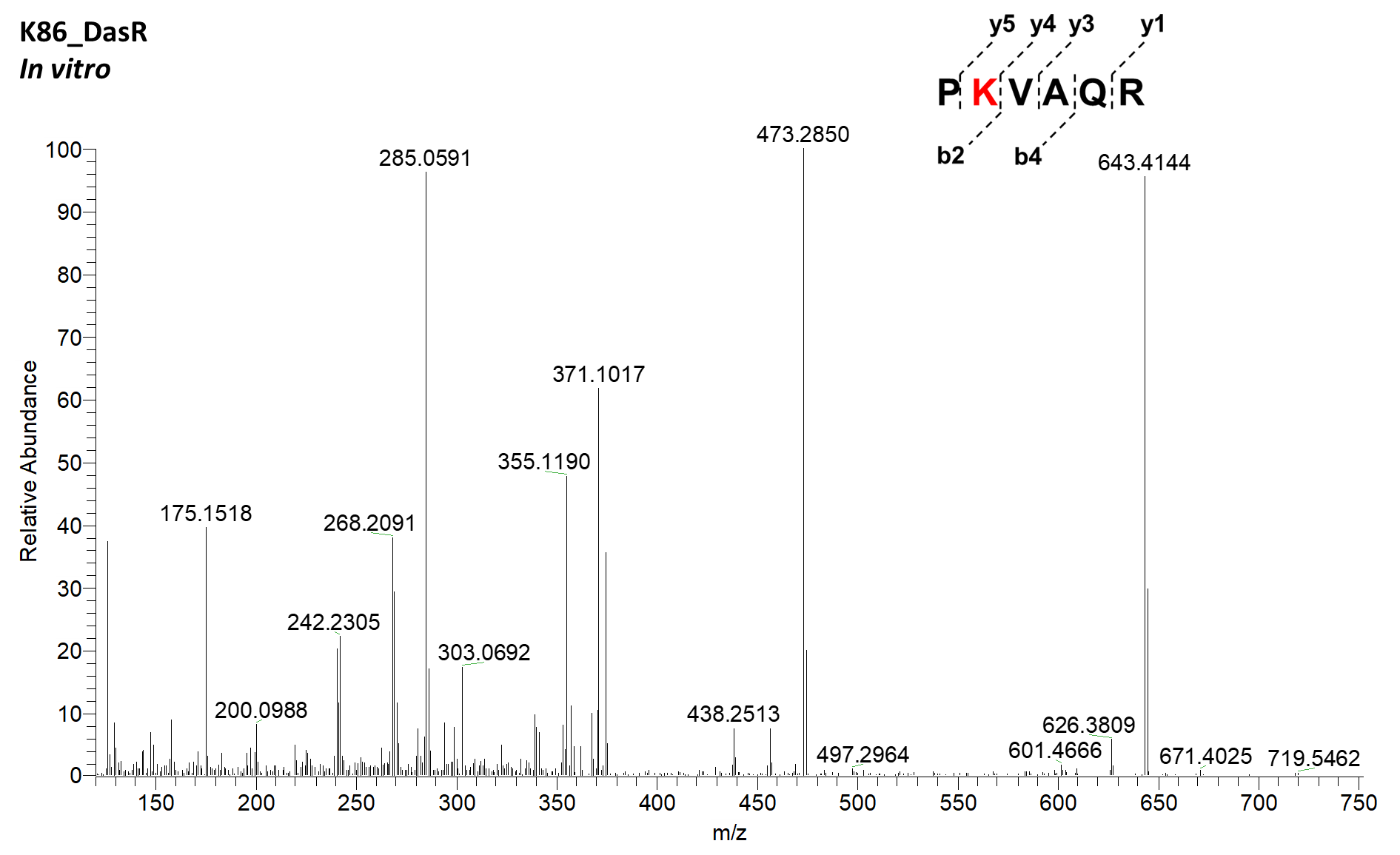
**

**Figure S7** **MS/MS spectra for the identification of K86 *in vitro* by LC-MS/MS analysis.**

**
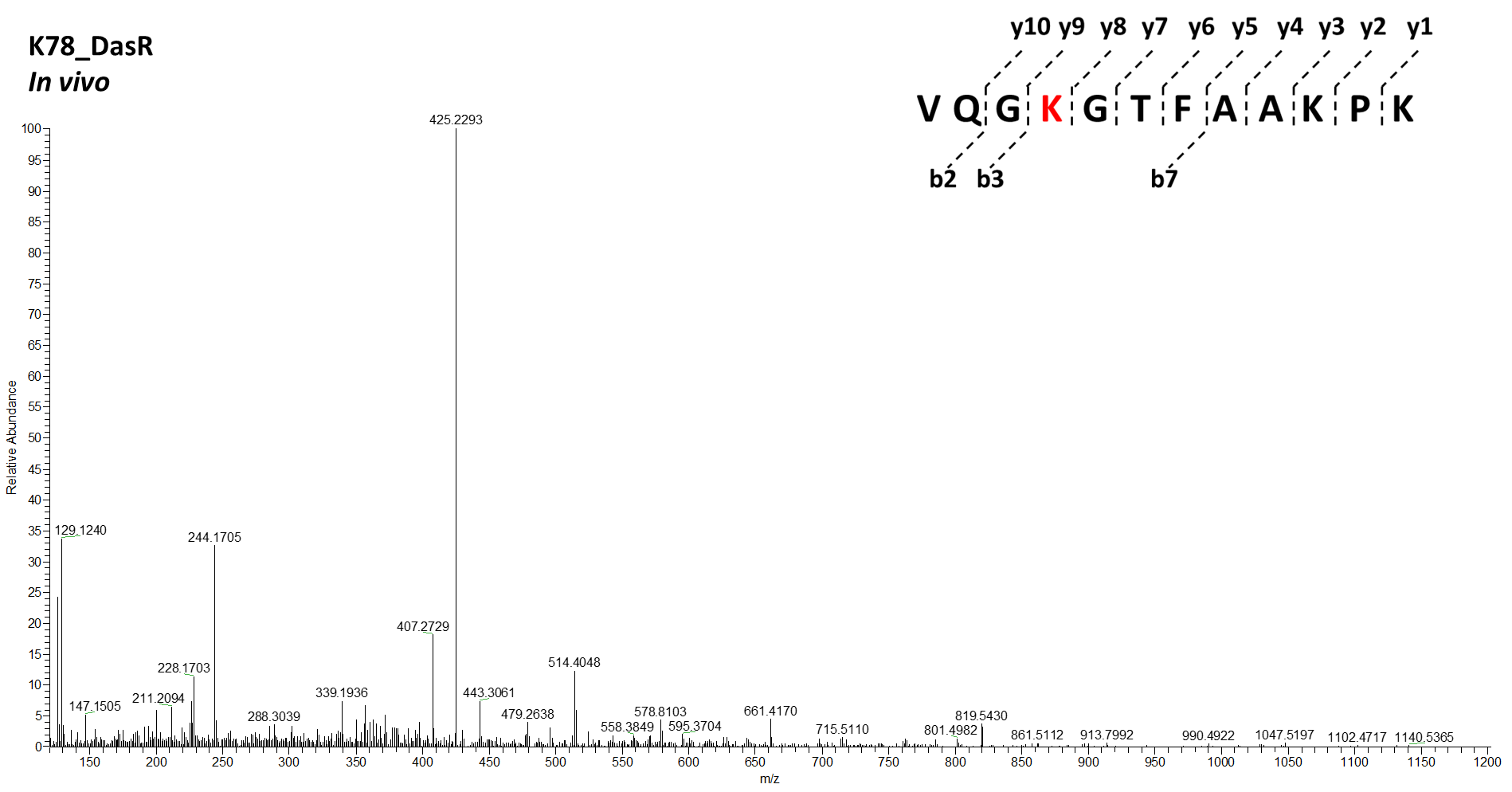
**

**Figure S8** **MS/MS spectra for the identification of K78 *in vivo* by LC-MS/MS analysis.**


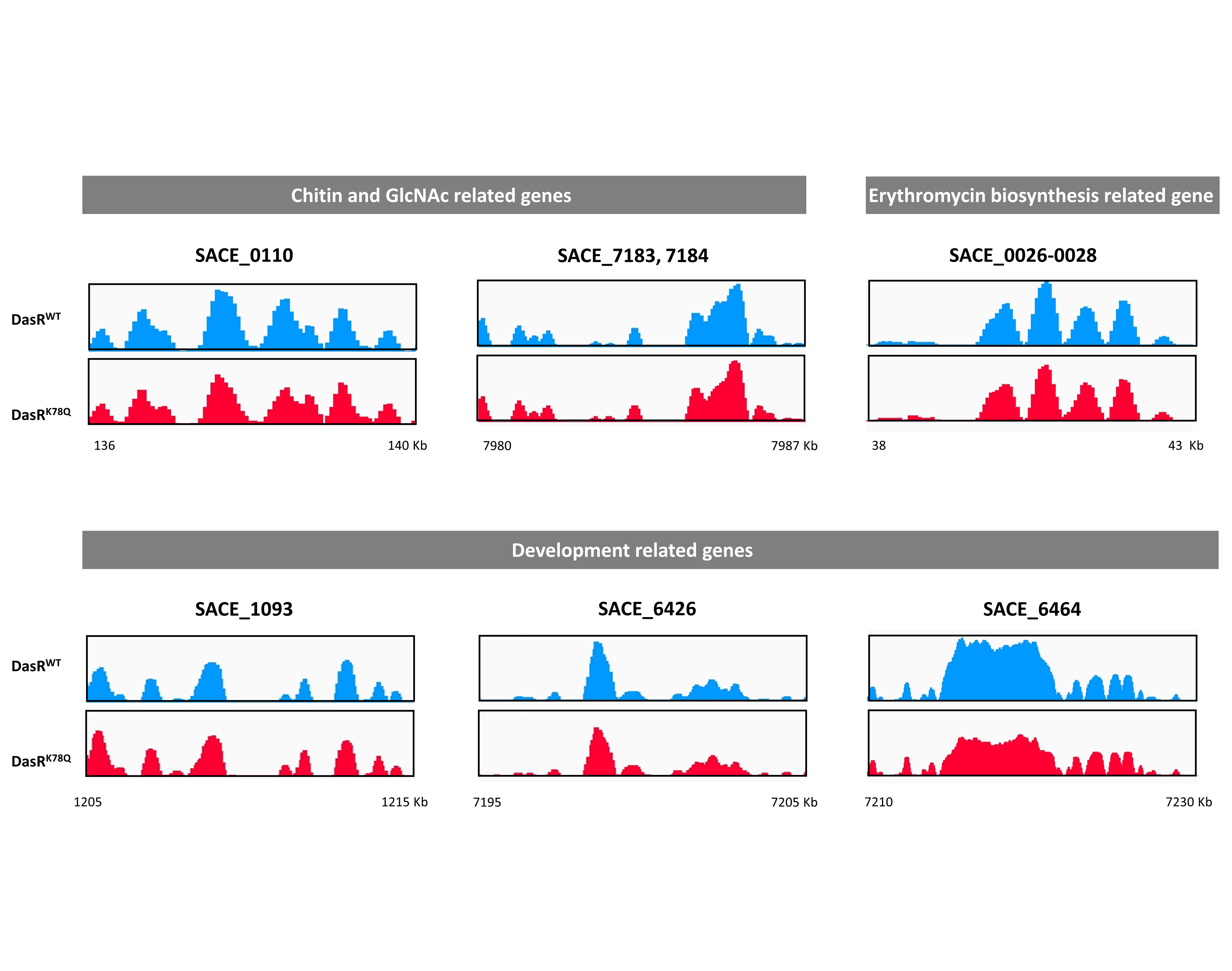


**Figure S9** Integrative Genomics Viewer (IGV) tracks showing ChIP-seq signals at the promoter regions of other targets in the indicated strains.


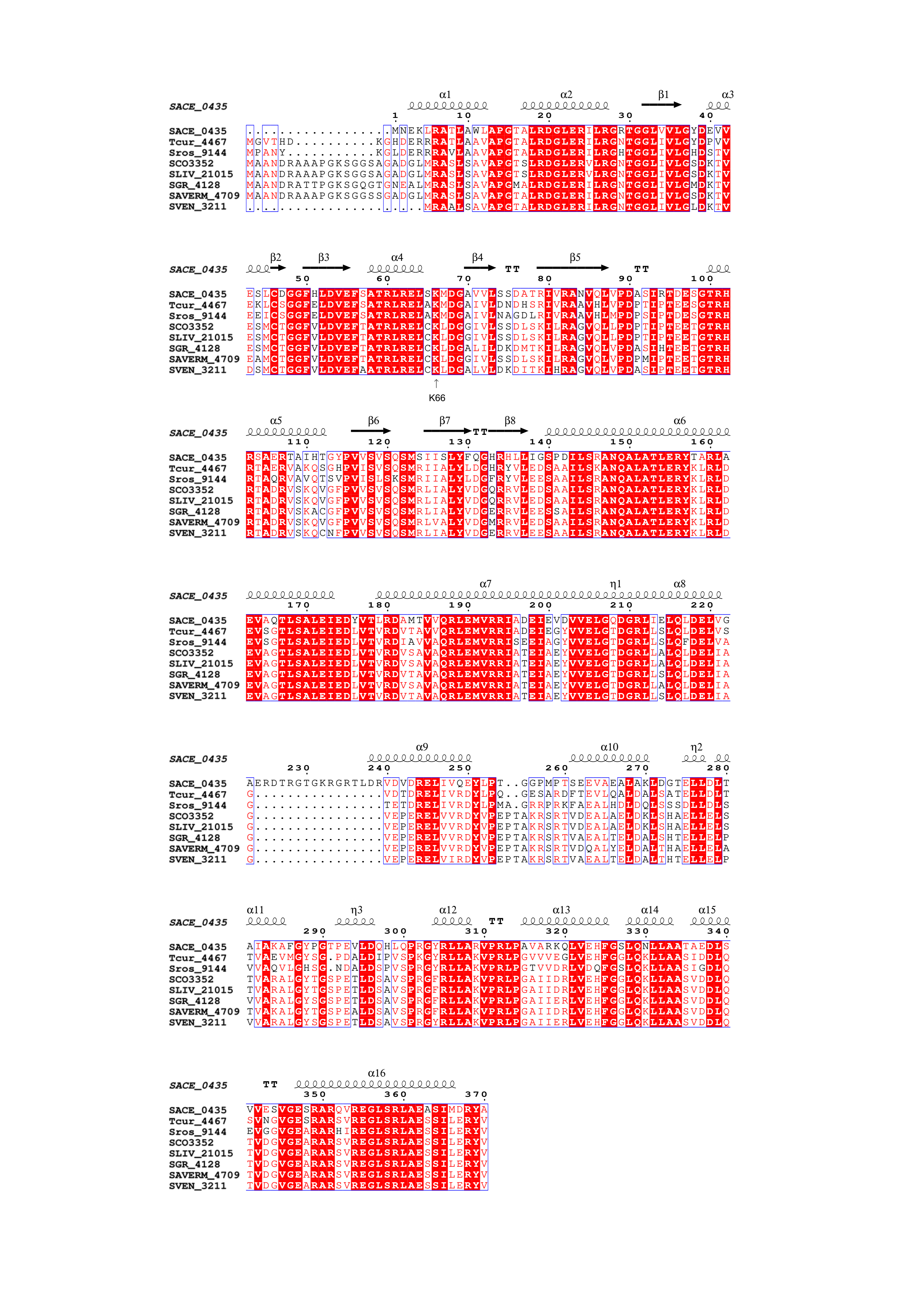


**Figure S10** **Sequence alignment of DisA proteins within actinobacteria.**


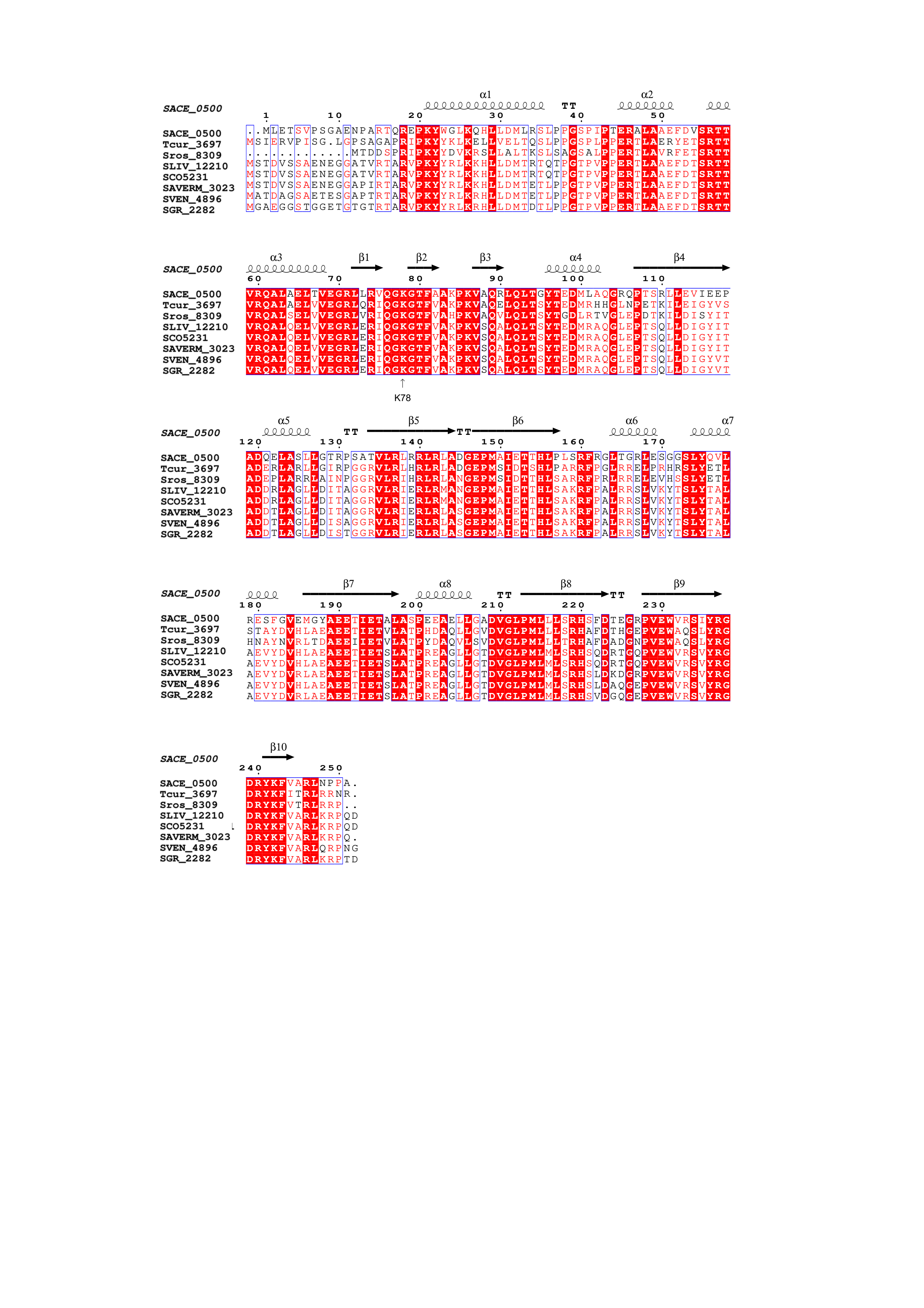


**Figure S11** **Sequence alignment of DasR proteins within actinobacteria.**


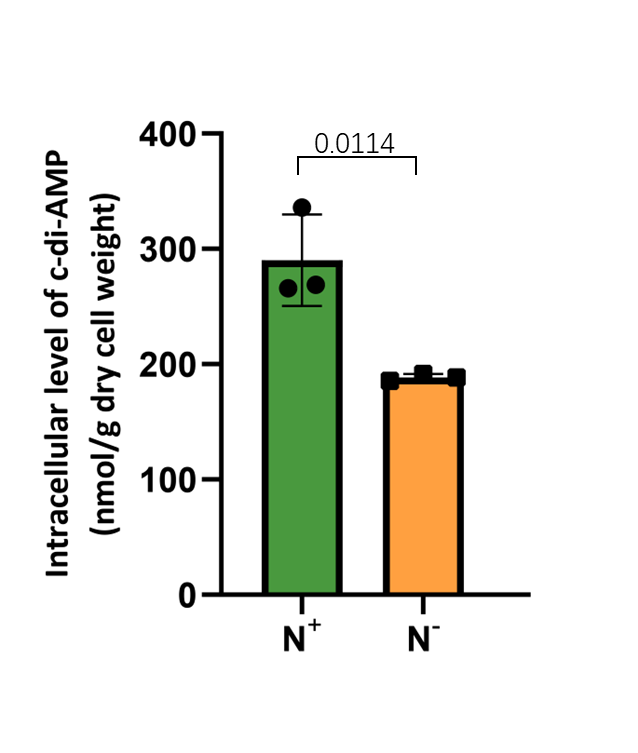


**Figure S12 Intracellular c-di-AMP levels in cell extracts of the *S. erythraea* WT strain grown in excess or limited nitrogen (N^+^ or N^-^) conditions**. The c-di-AMP concentrations of the samples were normalized to the dry cell weight. The error bars show the SDs of three independent experiments. A t test was used for statistical analysis.
